# Supplementary figures and images for: Single-cell transcription analysis of Plasmodium vivax blood-stage parasites identifies stage- and species-specific profiles of expression
Source: PLoS Biol. 2020 May 4;18(5):e3000711. doi: 10.1371/journal.pbio.3000711 (PMC7224573; doi:10.1371/journal.pbio.3000711)

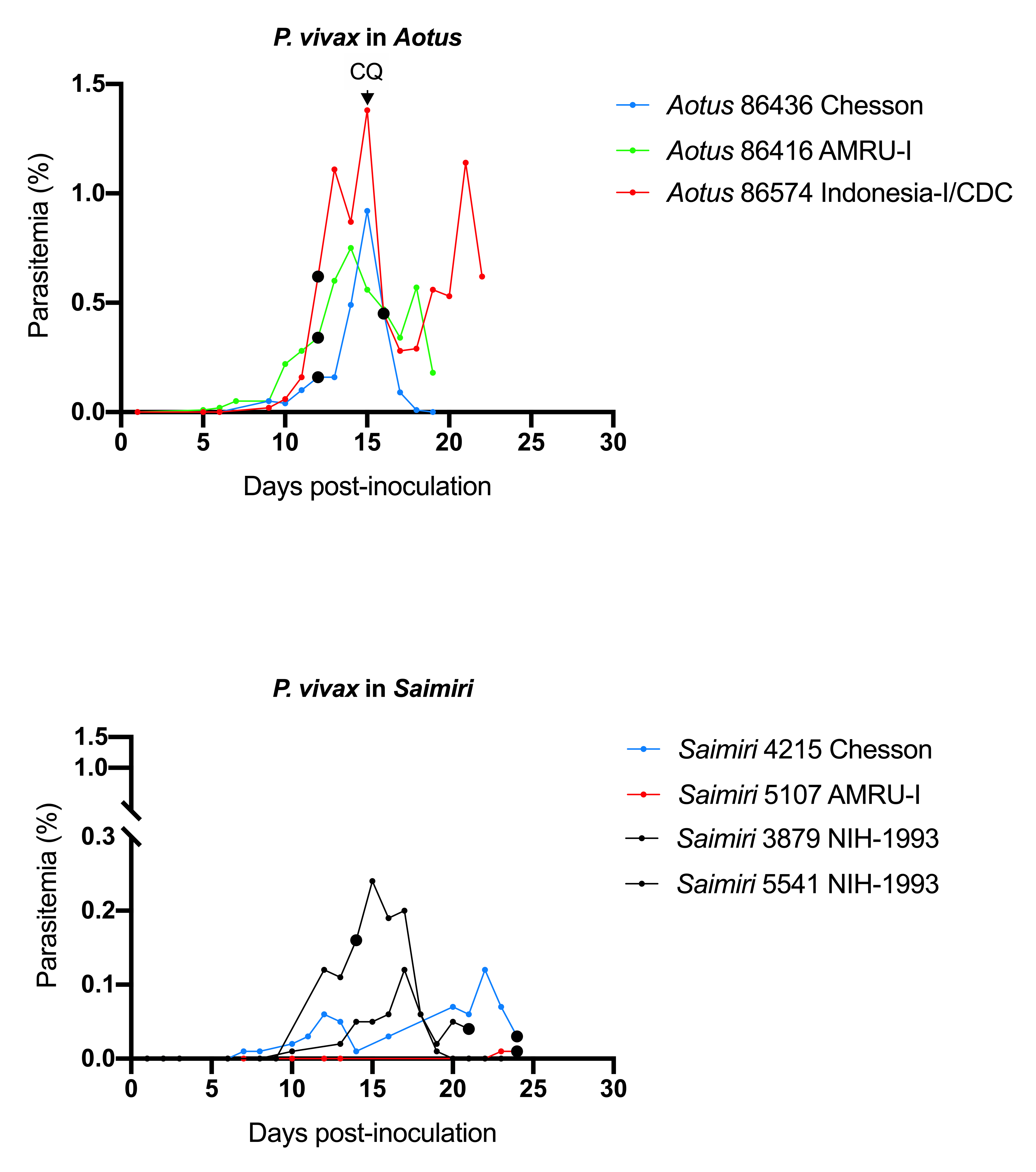

Supplement: S1 Fig — The graphs show the changes in parasitemia over time in Aotus (A) and Saimiri (B) monkeys infected with different strains of P. vivax. The large black circles represent, for each animal, the collection points of blood samples used for scRNA-seq library preparation. “CQ” indicates point of single oral administration of chloroquine to the animals (10 mg/kg for the Chesson and AMRU-I infections, 5 mg/kg for the Indonesia-I/CDC infection). Underlying data plotted in panels A and B are provided in S6 Data. scRNA-seq, single-cell RNA sequencing. (TIFF) [file pbio.3000711.s001.tiff]

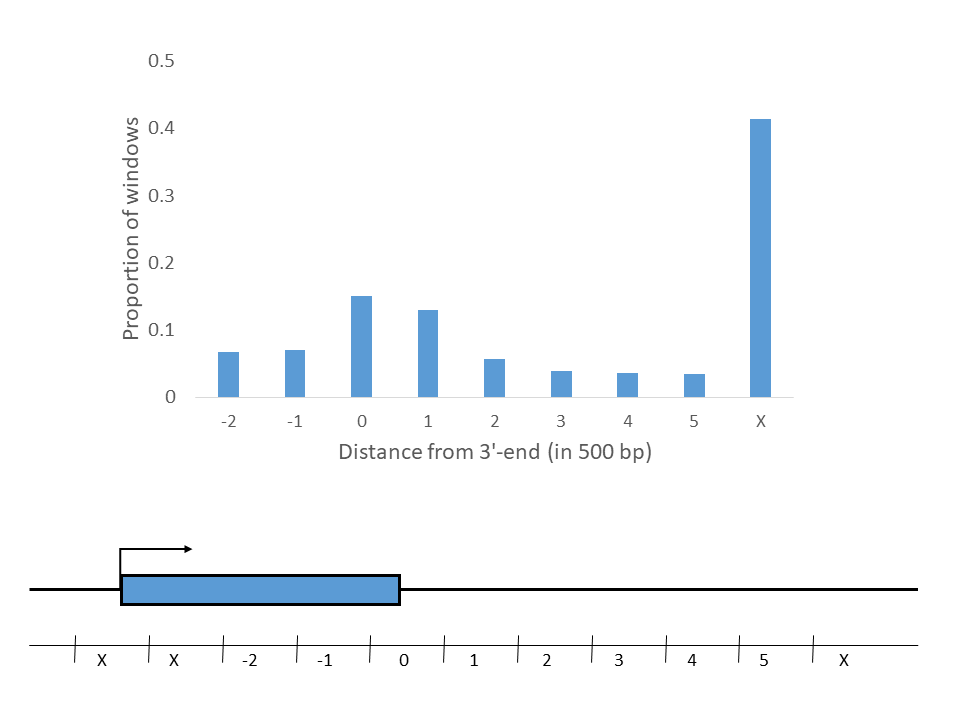

Supplement: S5 Fig — Each 500 bp window was annotated based on its distance from the 3’-end of the closest annotated gene. “0” corresponds to a window containing an annotated 3’-end, “1” through “5” indicate windows located 1 to 5 windows of 500 bp downstream of the annotated 3’-end, and “-2”/”-1” upstream of the 3’-end. “X” represents bins located outside of these regions. The annotation was performed independently for each DNA strand. The histogram on top shows the distribution of the windows containing scRNA-seq reads with regard to annotated genes. Underlying data are provided in S9 Data. bp, base pairs; scRNA-seq, single-cell RNA sequencing. (TIF) [file pbio.3000711.s005.tif]

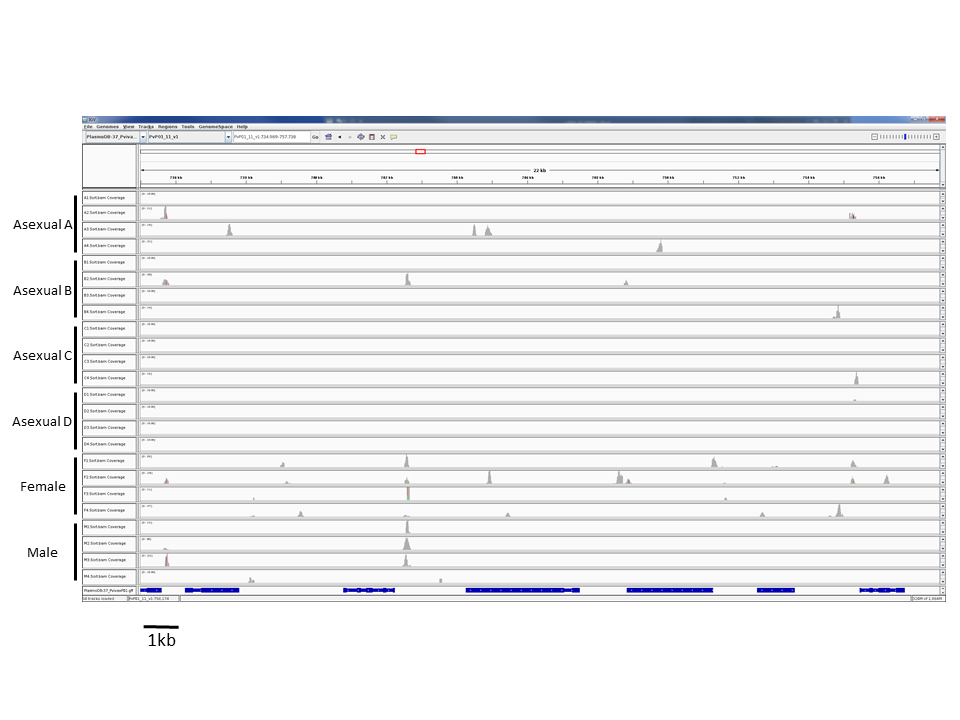

Supplement: S6 Fig — The figure shows approximately 20 kb on chromosome 11 between position 736,000 and 756,000. The blue bars at the bottom represent 7 annotated P. vivax genes in this region. The gray histograms represent the number of reads covering a given position for 24 single-cell transcriptomes generated from an infection of an Aotus monkey with AMRU-I. The first 16 rows correspond to asexual parasites along their developmental trajectories (by groups of 4), the next 4 rows represent female gametocytes, and the last 4 rows, male gametocytes. scRNA-seq, single-cell RNA sequencing. (TIF) [file pbio.3000711.s006.TIF]

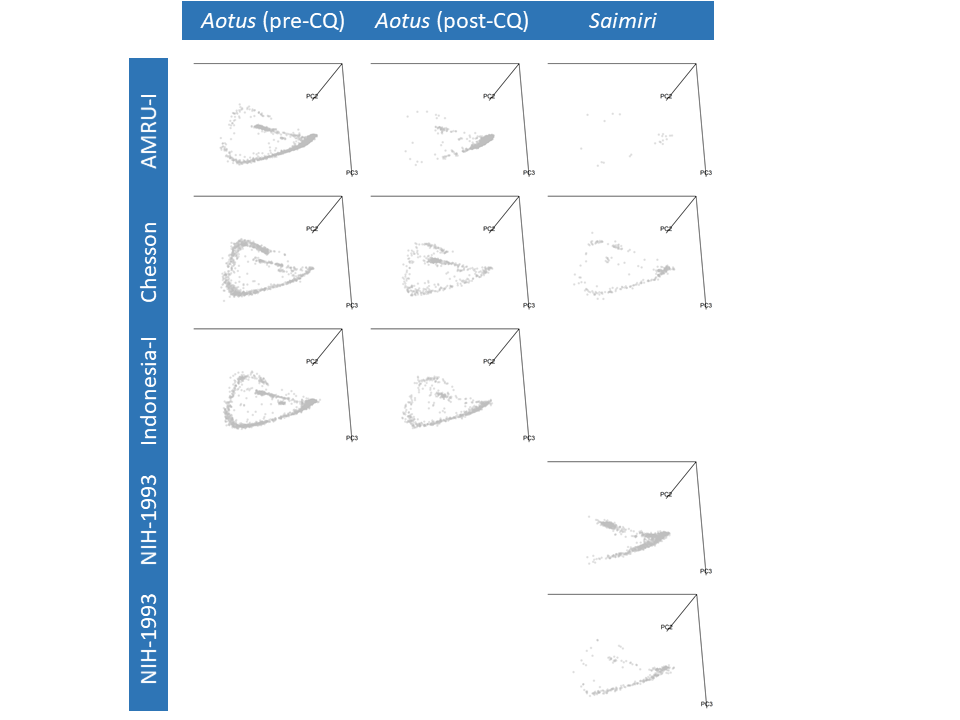

Supplement: S9 Fig — The figure shows PCA from all individual transcriptomes separated by blood samples. The rows correspond to the different strains of P. vivax used in this study, the columns blood samples collected from Aotus monkeys before and after chloroquine treatment (columns 1 and 2, respectively) or from Saimiri monkeys (column 3). Note that 2 different Saimiri monkeys were infected with the same strain of NIH-1993 (last 2 rows). Underlying data are provided in S10 Data. PCA, principal component analysis. (TIF) [file pbio.3000711.s009.tif]

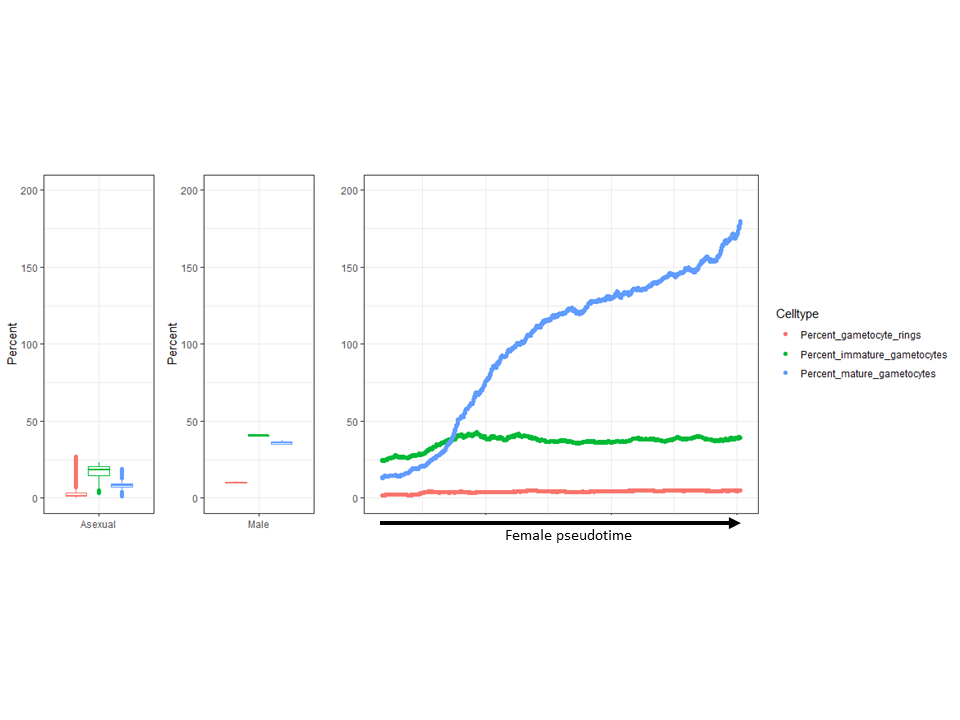

Supplement: S10 Fig — The panels show the average proportion of transcripts (y-axis, in mRNA molecules per 1,000) derived from P. vivax genes associated with GR (red), IG (green), and MG (blue) based on Obaldia and colleagues, 2018. The left and middle panels show the expression in asexual parasites and male gametocytes, respectively. The right panel displays the average expression of 50 female gametocytes organized according to their pseudotime (left, differentiating; right, fully differentiated). Underlying data are provided in S11 Data. GR, gametocyte ring; IG, immature gametocyte; MG, mature gametocyte. (TIF) [file pbio.3000711.s010.TIF]

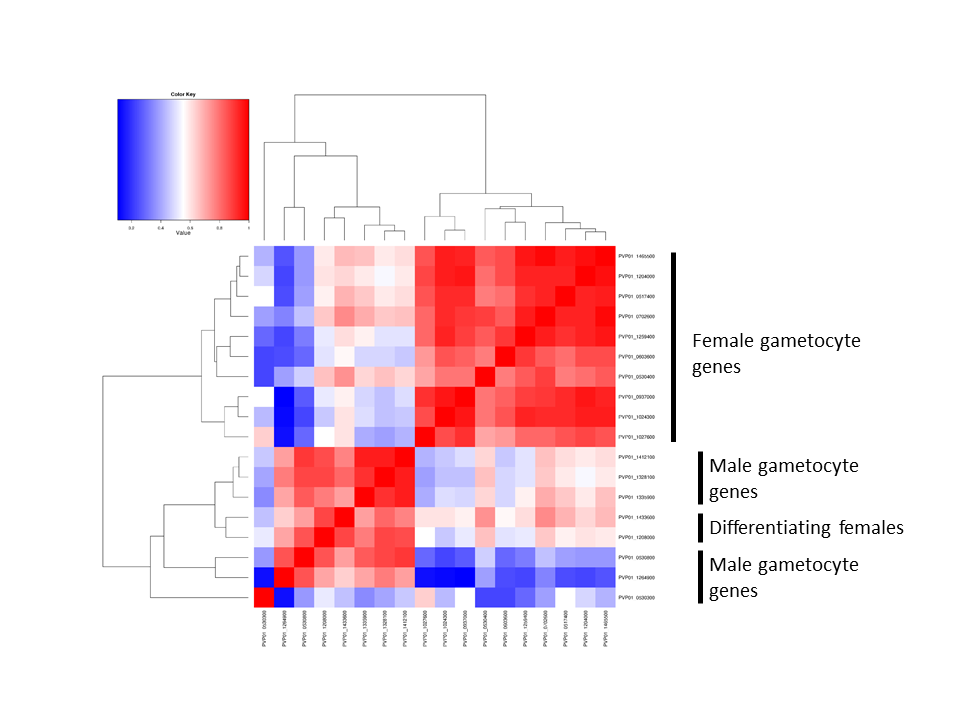

Supplement: S11 Fig — The heatmap shows the extent of gene expression correlation (Pearson’s R, in blue-red scale) between 2 gametocyte genes selected from scRNA-seq data across all Cambodian patients characterized in Kim and colleagues, 2019. The bordering trees show the result of unsupervised clustering of these genes according to their gene expression patterns. Underlying data are provided in S12 Data. scRNA-seq, single-cell RNA sequencing. (TIF) [file pbio.3000711.s011.TIF]

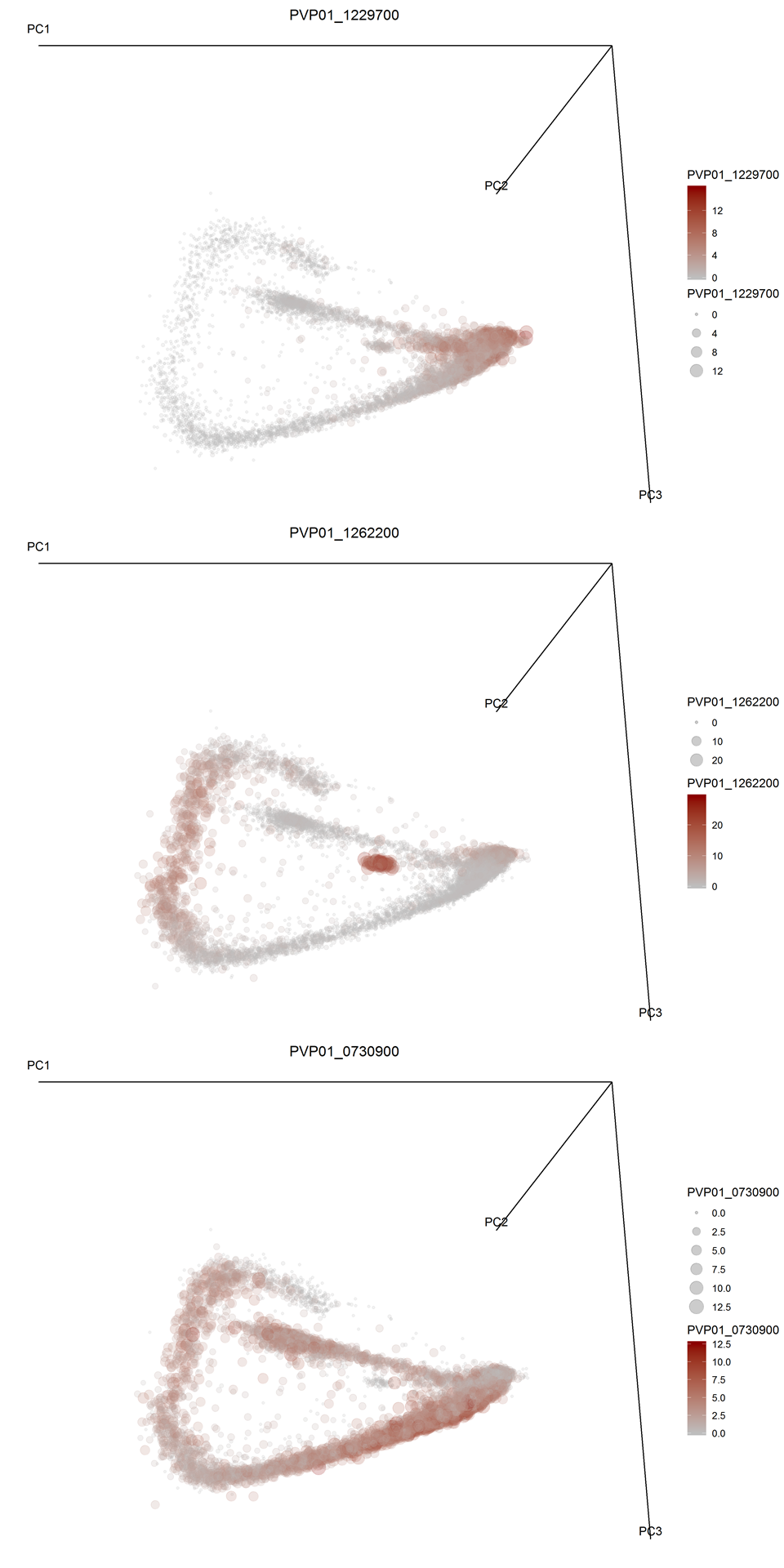

Supplement: S12 Fig — The figures show PCA from all P. vivax transcriptomes colored according to the expression level of, from top to bottom, 2 genes used in RDTs, lactate dehydrogenase (PVP01_1229700) and fructose 1,6-bisphosphate aldolase (PVP01_1262200), and one gene almost ubiquitously expressed (profilin or PVP01_0730900). Each dot represents one single-cell parasite and is colored according to the expression level of that transcript (in red scale). Underlying data are provided in S13 Data. PCA, principal component analysis; RDT, rapid diagnostic test. (TIF) [file pbio.3000711.s012.tif]

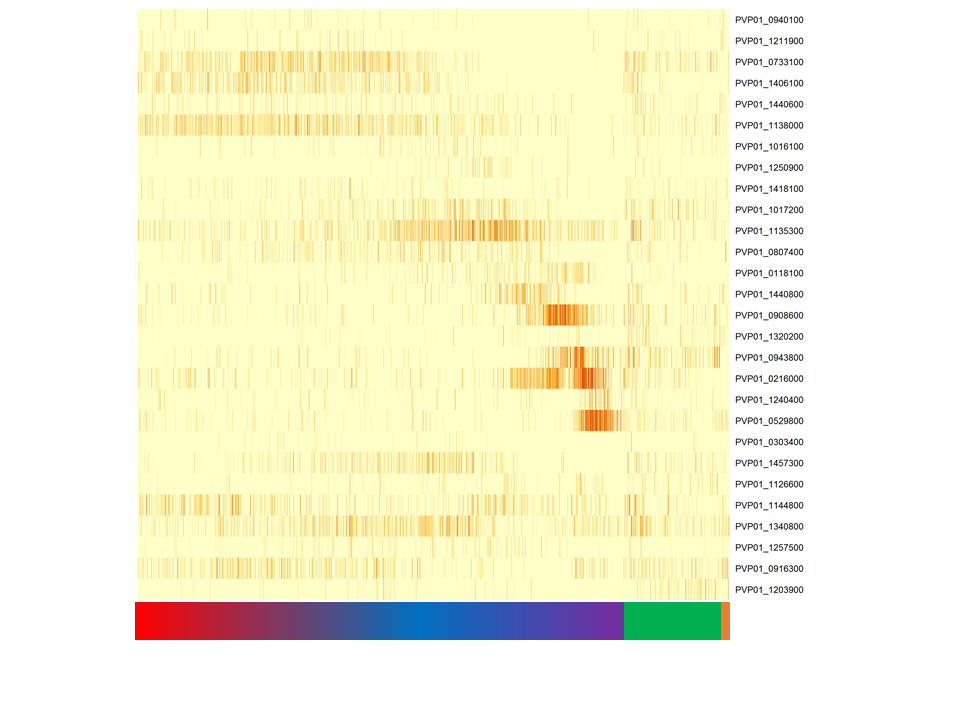

Supplement: S13 Fig — Variations in the expression levels of AP2 genes according to the parasite development. Each row represents a different annotated AP2 gene and each vertical bar an individual P. vivax parasite ranked along the x-axis based on its pseudotime. The color of each vertical bar represents the expression level of a specific transcript from nondetected (yellow) to highest expression (in red) in yellow-to-red scale. The color bar under the heatmap shows the assignment of the parasites into different morphological stages (red, trophozoites; blue and purple, schizonts; green, female gametocytes; orange, male gametocytes). Underlying data are provided in S14 Data. (TIF) [file pbio.3000711.s013.tif]

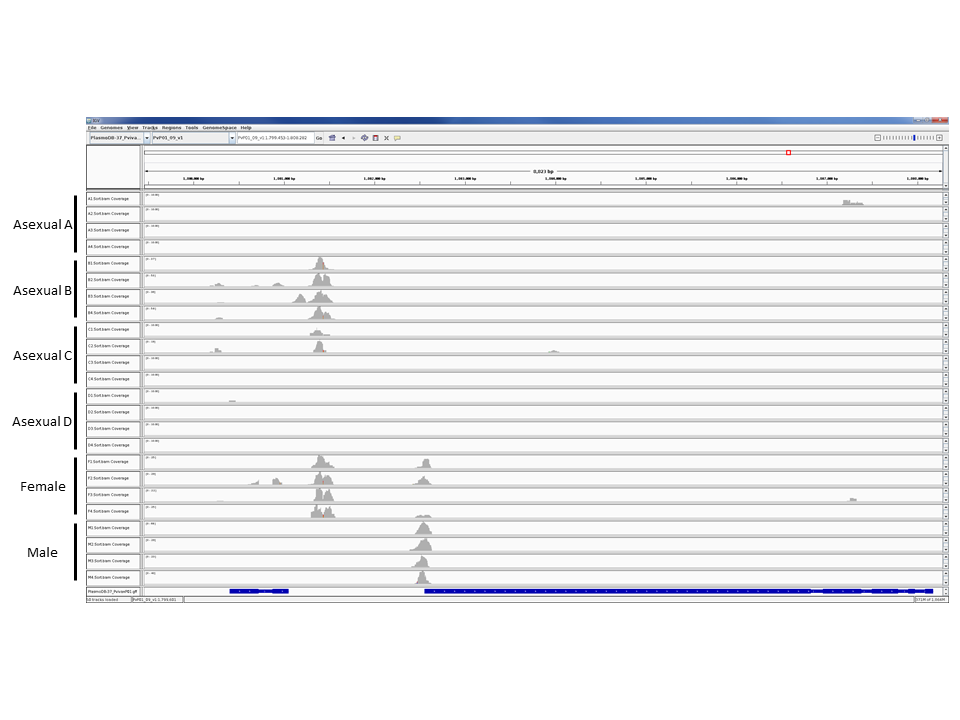

Supplement: S14 Fig — The figure shows scRNA-seq data from 24 individual parasites for a region of approximately 10 kb on chromosome 9 containing 2 annotated P. vivax genes, PVP01_0941100 (left blue bar) and PVP01_0941200 (right blue bar), both encoded on the + strand. The scRNA-seq sequences generated from for asexual parasites (rows 1–16) map close to the annotated 3’-end of PVP01_0941100, whereas those generated from male gametocytes (last 4 rows) map more than 1 kb downstream. Female gametocytes (rows 17–20) displayed an intermediate profile. This pattern is consistent with the presence of 2 different 3’-UTRs for PVP01_0941100 (although it is also possible that the second peak corresponds to the 3’-UTR of an unannotated P. vivax gene). scRNA-seq, single-cell RNA sequencing; UTR, untranslated region. (TIF) [file pbio.3000711.s014.TIF]

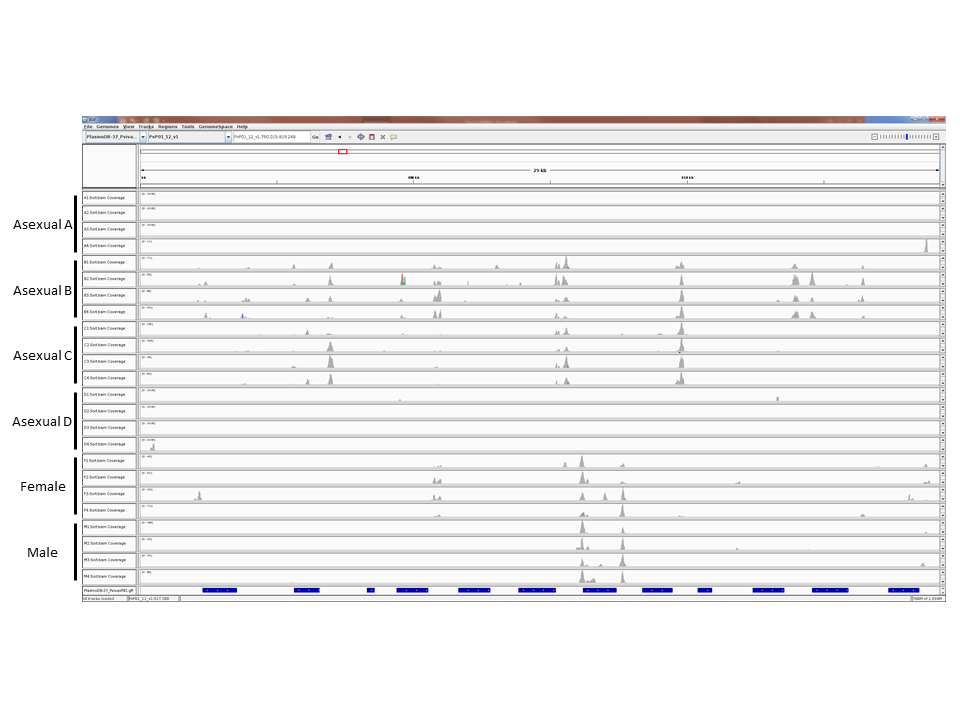

Supplement: S15 Fig — The figure shows examples of scRNA-seq data across approximately 30 kb of chromosome 12 containing a cluster of MSP7-like genes (blue bars at the bottom). Note that most MSP genes are specifically expressed in late asexual parasites (rows 5–12) but turned off in very late schizonts (rows 13–16), whereas other MSP7-like genes are uniquely expressed in female and male gametocytes (row 17–24). MSP, merozoite surface protein. (TIF) [file pbio.3000711.s015.tif]

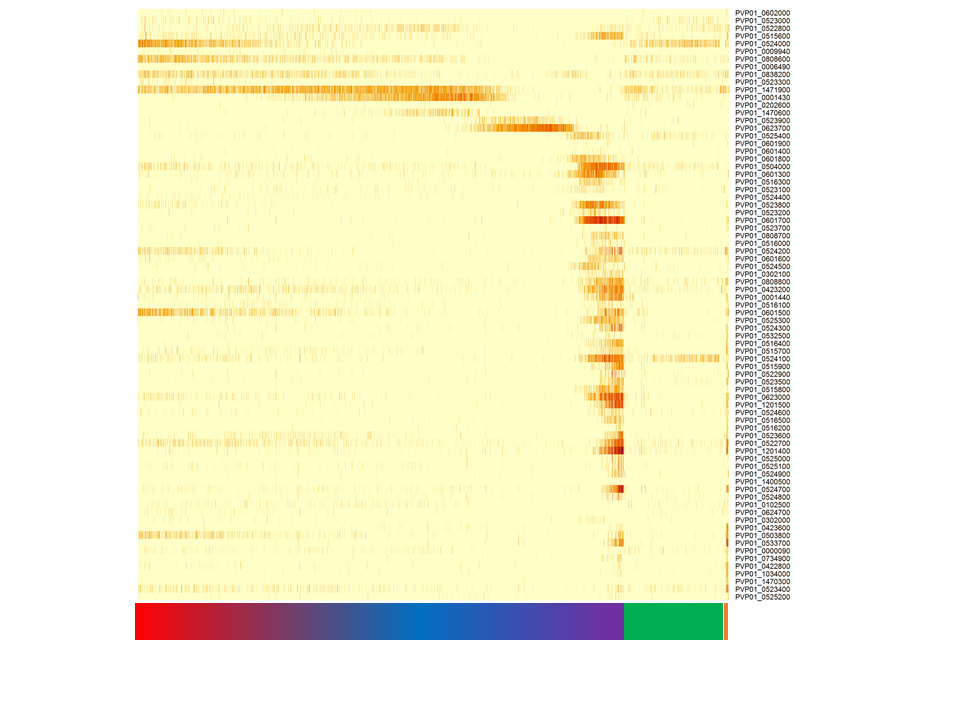

Supplement: S16 Fig — Variations in the expression levels of members of the PHIST multigene family according to the parasite development. Each row represents a different annotated PHIST gene, and each vertical bar an individual P. vivax parasite ranked along the x-axis based on its pseudotime. The color of each vertical bar represents the expression level of a specific transcript from nondetected (yellow) to highest expression (in red) in yellow-to-red scale. The color bar under the heatmap shows the assignment of the parasites into different morphological stages (red, trophozoites; blue and purple, schizonts; green, female gametocytes; orange, male gametocytes). Underlying data are provided in S15 Data. PHIST, plasmodium helical interspersed subtelomeric. (TIF) [file pbio.3000711.s016.tif]

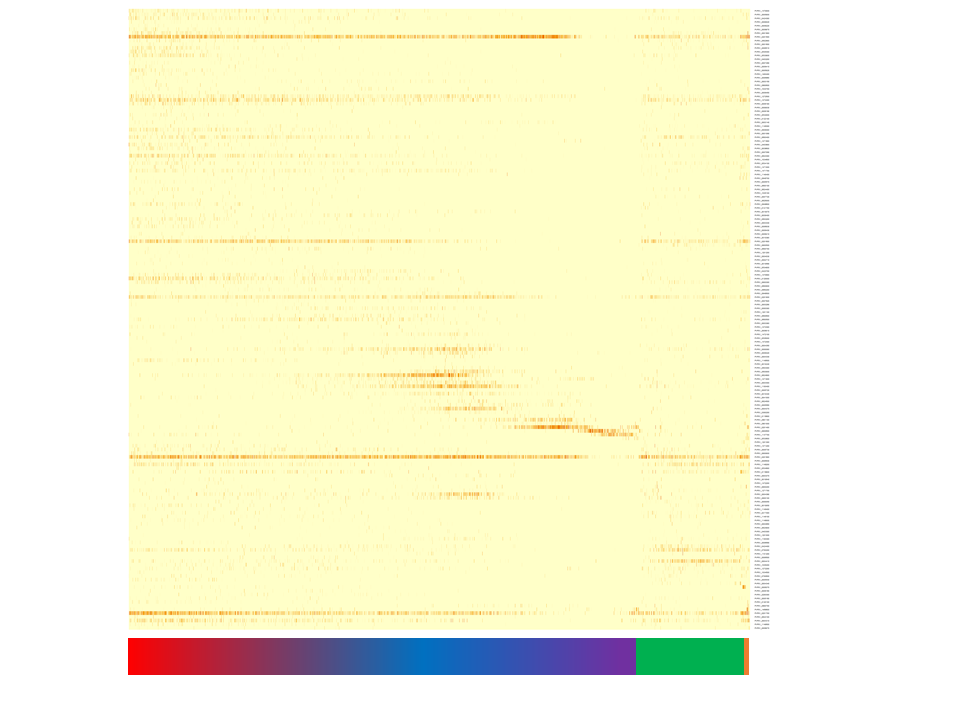

Supplement: S17 Fig — Variations in the expression levels of PIR genes according to the parasite development. Each row represents a different annotated PIR gene and each vertical bar an individual P. vivax parasite ranked along the x-axis based on its pseudotime. The color of each vertical bar represents the expression level of a specific transcript from nondetected (yellow) to highest expression (in red) in yellow-to-red scale. The color bar under the heatmap shows the assignment of the parasites into different morphological stages (red, trophozoites; blue and purple, schizonts; green, female gametocytes; orange, male gametocytes). Underlying data are provided in S16 Data. (TIF) [file pbio.3000711.s017.tif]

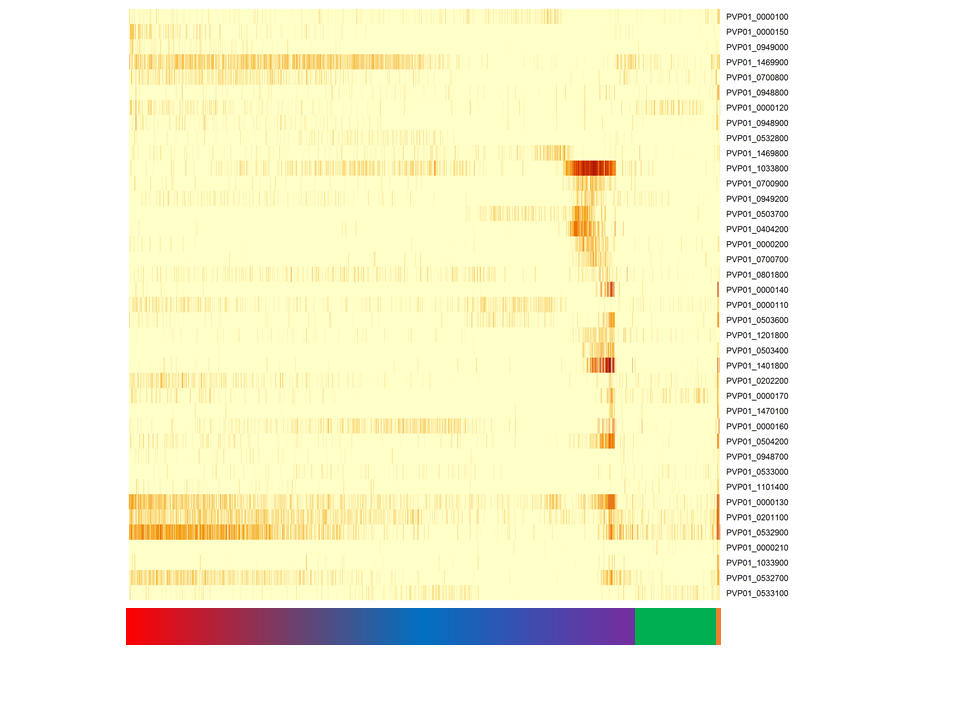

Supplement: S18 Fig — Variations in the expression levels of tryptophan-rich antigen genes according to the parasite development. Each row represents a different annotated tryptophan-rich antigen gene and each vertical bar an individual P. vivax parasite ranked along the x-axis based on its pseudotime. The color of each vertical bar represents the expression level of a specific transcript from nondetected (yellow) to highest expression (in red) in yellow-to-red scale. The color bar under the heatmap shows the assignment of the parasites into different morphological stages (red, trophozoites; blue and purple, schizonts; green, female gametocytes; orange, male gametocytes). Underlying data are provided in S17 Data. (TIF) [file pbio.3000711.s018.tif]

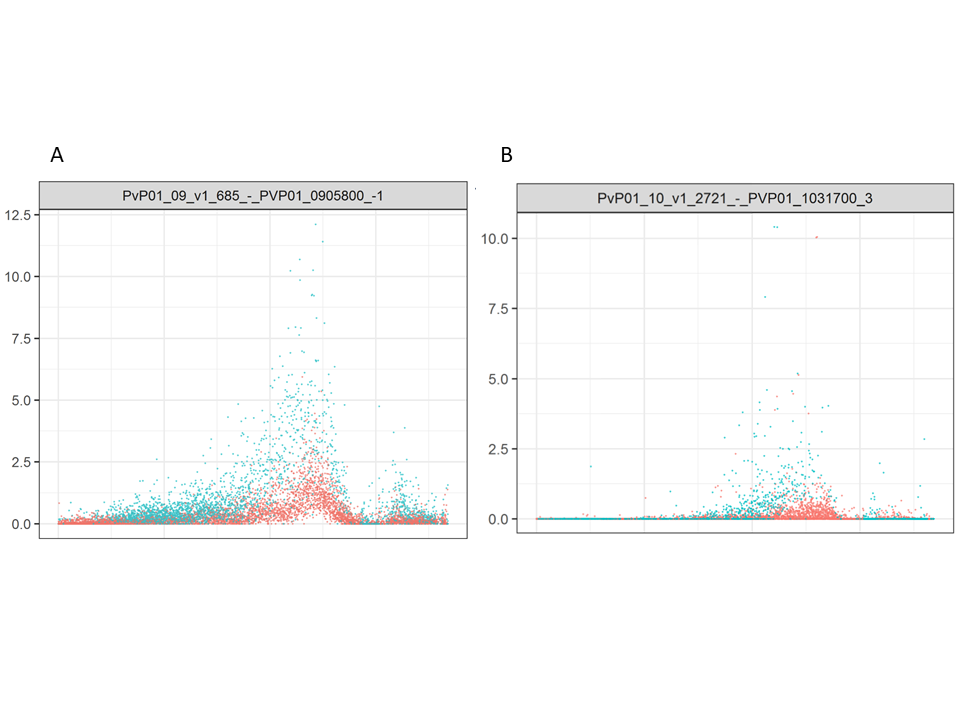

Supplement: S20 Fig — (A) Expression of histone H4 (PVP01_0905800) in individual parasites organized along their developmental pseudotime rank (on the x-axis) and colored according to the chloroquine treatment (red, before treatment; blue, after treatment). Only parasite transcriptomes generated from Aotus infections are represented. (B) Expression of the exported protein PVP01_1402200 in individual parasites organized along their developmental pseudotime rank and colored according to the host species (red, Aotus; blue, Saimiri). The y-axis indicates the number of unique reads mapped to these windows in each parasite (in reads per 1,000). Underlying data are provided in S19 Data. (TIF) [file pbio.3000711.s020.TIF]

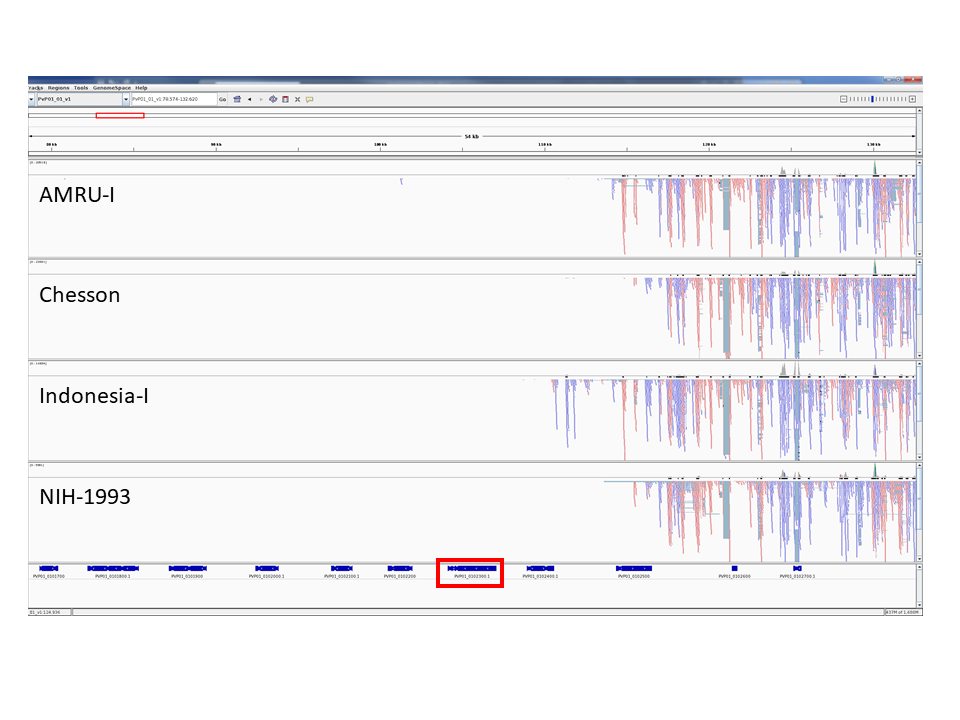

Supplement: S21 Fig — The figure shows all the reads from each P. vivax strain mapped to a 50-kb region surrounding DBP2 (PVP01_0102300, highlighted in red). Virtually no reads mapped in the first 110 kb of this chromosomal arm (including DBP2) possibly indicating a deletion of this region in these monkey-adapted strains. Note that the boundary of this deletion might differ between strains (as shown by the location where read mapping resumed). (TIF) [file pbio.3000711.s021.tif]

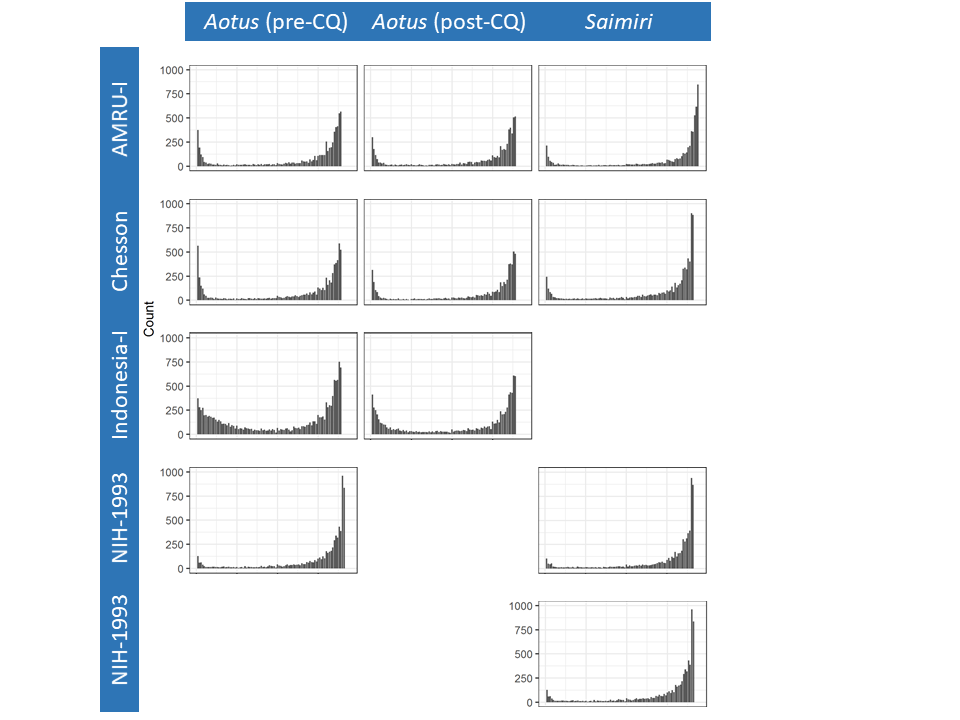

Supplement: S22 Fig — The figure shows reference allele frequency plots generated from each blood sample. Each plot shows the proportion of reads carrying the reference allele (x-axis, for 0 on the left to 1 on the right) for each position sequenced at >20X (y-axis). The rows correspond to the different strains of P. vivax used in this study, the columns blood samples collected from Aotus monkeys before and after chloroquine treatment (columns 1 and 2, respectively) or from Saimiri monkeys (column 3). Note that 2 different Saimiri monkeys were infected with the same strain of NIH-1993 (last 2 rows). Underlying data are provided in S20 Data. (TIF) [file pbio.3000711.s022.tif]
