# Supplementary material for: Single-cell transcription analysis of Plasmodium vivax blood-stage parasites identifies stage- and species-specific profiles of expression
Source: PLoS Biol. 2020 May 4;18(5):e3000711. doi: 10.1371/journal.pbio.3000711 (PMC7224573; doi:10.1371/journal.pbio.3000711)
Supplement: S1 Text — Description of the assessment of clonality and the number of genetic differences between strains. (DOCX) [file pbio.3000711.s029.docx]

**S1 Text**

**Genetic Analyses**

*Assessment of clonality*

To assess whether the strains used to infect the New World monkeys contained multiple, genetically different clones, we first examined, separately for each infection, allelic variations at each nucleotide position sequenced by more than 20 reads overall (combining the reads from all parasites from one sample). Across the entire genome, the reads either all carried the reference allele or all carried the alternative allele (**S22 Figure**), consistent with the presence of a single main clone in each infection ^43^ (though the sensitivity of these analyses cannot exclude the presence of genetically different clones at a prevalence of <5%).

*Strain authentication and genetic differences between strains*

To verify the identity of the cryopreserved *P. vivax* parasites, we examined single nucleotide differences between samples across 2,279,246 nucleotides sequenced at >20X in all strains (using directly the sequence information from the scRNA-seq data). As expected, we observed no genetic differences between the *P. vivax* strains sequenced from the same *Aotus* monkeys before and after chloroquine treatment, validating our approach and the low false positive rate of the genotyping calls (**S6 Table**). Similarly, very few differences were observed between the genomes of the same parasite strain propagated in two different hosts, though slightly more differences were observed in AMRU-I, possibly indicating the accumulation of substitutions during the propagation of this strain in different lines. On the other hand, 1,586-2,377 nucleotide positions differed between any two pairs of isolates (**S6 Table**). Finally, we confirmed that the NIH-1993 parasites used here were derived from, and highly genetically similar to, the Salvador-I strain of *P. vivax* by mapping all the reads from this strain to the reference *P. vivax* genome ^73^ which only revealed 386 putative nucleotide differences between these two genomes out of 10,311,819 bp sequenced at >20X.
