# Supplementary material for: Single-cell transcription analysis of Plasmodium vivax blood-stage parasites identifies stage- and species-specific profiles of expression
Source: PLoS Biol. 2020 May 4;18(5):e3000711. doi: 10.1371/journal.pbio.3000711 (PMC7224573; doi:10.1371/journal.pbio.3000711)
Supplement: Attachment — Submitted filename: ResponseReviewers_DS010320.docx [file pbio.3000711.s050.docx]

**We would like to thank the editor and the reviewers for their positive assessment of our manuscript and their constructive comments and suggestions, which have significantly improved our study. We apologize for the delay in submitting this revised manuscript but addressing the reviewers’ comments required numerous analyses that took much longer than we had anticipated. We have now modified our manuscript to address the reviewers’ concerns and have included their suggestions in the main text plus extensively expanded the supplemental information. We present below our point-by-point answer to all of the reviewer and editor comments (the page numbers refer to the revised manuscript).**

Reviewer #1

The current manuscript describes application of a single-cell sequencing approach to characterising stage specific transcriptomes for blood-stages of Plasmodium vivax taken from experimental infected primates. The work is very interesting and the potential identification of stage-specific markers, particularly for gametocyte stages, is of high value.

**We thank the reviewer for this kind assessment of our work and its value to advance understanding of *Plasmodium vivax* biology.**

Major comments:

1. I have some significant concerns about the normalization strategy used in the study. The authors have mapped the data using standard and appropriate tools and removed PCR duplicate reads, which is appropriate and considered the current gold-standand approach for scRNA-seq data. However, to normalize the data, they have discarded low and high coverage outliers and then normalized their read count data (binned by mapping window) based on the total reads generated for the cell. This approach is essentially the "transcripts per million" transformation used in bulk RNA-seq analyses. This is fine, but even for bulk data, it is necessary to use more sophisticated normalization methods (e.g., TMM or UQ normalization) prior to TPM calculation, which are more robust to large differences in sequencing depth and other potential biases in RNA-seq data.

For scRNA-seq, the preferred approach is to include quantitative spike-in controls to support data normalization. In the absence of this, it is necessary to use normalization strategies specifically suited to scRNA-seq data. Removal of high and low coverage outlier cells would help with this, but it is not considered enough on its own. A recent study hosted on BioRxiv (<https://www.biorxiv.org/content/early/2019/03/19/583013.full.pdf>) systematically evaluates scRNA-seq analytical approaches and highlights the appropriate data normalization as the most critical step in this process. In my opinion, the authors need to use a more robust normalization strategy, such as SCnorm (<https://www.ncbi.nlm.nih.gov/pmc/articles/PMC5473255/>) before reappraising the other steps in their study. I realise this is inconvenient and time-consuming, but I don't think it is possible to confidently make major conclusions based on the current analytical approach.

**We thank the reviewer for this comment and for providing detailed literature references. We have invested several weeks running different normalization approaches and testing their impact on the conclusions of our study. We primarily focused here on the results from *scran*, which is believed to perform best for most experimental setups according to the systematic evaluation by Vieth *et al*. 2019.**

**For the analysis of differential gene expression, we have now incorporated the scaling factors calculated by *scran* as suggested by the reviewer and recommended by Vieth et al., 2019 (see our response to the reviewer’s next comment). Regarding the clustering analysis, our comparisons showed that the profiles obtained after *scran* normalization were similar to those from our initial analysis (Figure A below), although with a “less tight” cell clustering and a loss of definition of the male gametocytes.**

|  |  |
| --- | --- |
| **Figure A. Principal component analysis of the *P. vivax* scRNA-seq data using our previous analysis (left) or the normalized data using *scran* (right).** | |

**In *scran*, the calculation of the scaling/size factors for each gene relies on prior grouping of “comparable cells” into clusters. Such crude clustering prior to the normalization efficiently handles discrete populations of cells (as typical of most human tissues) but might not perform as well when the gene expression varies gradually among cells, as in the case of the *Plasmodium* intraerythrocytic cycle. In addition, genes specifically expressed in a minority of the cells, such as the male gametocytes that represent ~1% of all cells, might be disproportionally underweighted (even though we assigned the minimal cluster size to 50 in our analysis). We should note that the proportion of variance explained by the major principal components in the PCA appeared much greater after *scran* normalization. However, this is an artefact of selecting only the transcripts with the highest biological variance to calculate the PCs in *scran* and does not accurately represent the data. Given these concerns and the overall similarity between the profiles obtained with and without normalization, we would prefer to display the results of the PCA using only the library size normalization as we performed initially. An advantage of this approach is that the read counts are directly proportional to number of mRNA molecules derived from each gene and are therefore more easily interpretable.**

2. Following from this, the authors describing using edgeR for paired analyses of their data. This is not described in detail, but I take this to mean for differential gene expression analyses. If so, the details of these analyses need to be provided (What significance threshold was used? Did the authors used a Fischer's test or a linear model (i.e., Limma) for differentational calling?). Further and more importantly, EdgeR is a robust package for RNAseq and works very well for bulk RNAseq data, but it was not designed for scRNA-seq data specifically. In the absence of control spike-ins, it is critical that the data be appropriately and robustly normalized before using EdgeR (as noted above). A detailed comparative analysis of methods for calling differentially transcribed genes in scRNA-seq data can be found here, for example, doi:10.1038/nmeth.4612 and highlights other important steps including determining dropout rates and imputing missing gene values. For a example of specific applications in Plasmodium, the authors could also look at <https://www.sciencedirect.com/science/article/pii/S0014482718306438>, which used an Upper-Quartile normalization approach coupled with an scRNA-seq aware differential expression caller (SCDE). Reid et al, eLife, 2018 provides another good example and includes a approach for imputing missing gene data, which is an important step (<https://www.ncbi.nlm.nih.gov/pmc/articles/PMC5871331/>).

**We agree and thank the reviewer for these constructive comments. We have now modified our pipeline for the differential gene expression analysis according to these suggestions. Briefly, we use the scaling factors calculated by *scran* and conduct statistical testing using the zinbwave package (which now replaces the zingeR package recommended by Vieth *et al.*, 2019). Paragraphs in the Methods (on page 7) and Results (on pages 15-16) sections have been modified accordingly.**

3. Results - page 8 - "Although accumulation of specific P. vivax stages has been reported in host tissues such as bone marrow the patterns, extent, and mechanisms of sequestration differ substantially from those of P. falciparum, and in many P. vivax infections, young as well as mature asexual and sexual stages can be found simultaneously in the circulation 27,40. Consistent with these observations, principal component analysis of the individual transcriptomes revealed distinct populations of blood stage parasites." - I'm not sure about the connection between the first and second sentence here. I understand the idea that P. vivax differs from P. falciparum in tissue sequestration patterns, but I don't see the direct connection to the distinct transcriptomic populations in the blood-stage data. Specifically, how do the authors differentiate that from simply different stage-specific transcriptomes? Do these population clusters differ markedly from the clusters seen in currently published scRNA-seq datasets for P. falciparum.

**We have now edited this sentence on page 9 to improve clarity. Since there is little sequestration of *P. vivax*-infected erythrocytes, a full range of life cycle stages (mature and immature, sexual and asexual) are often found in infected blood samples. This diversity is reflected by the diversity of transcriptome profiles obtained by scRNA-seq as shown in Figure 1A or Figure A above. We have now confirmed that these different transcriptomes correspond to different developmental stages by comparing the *P. vivax* scRNA-seq profiles with those generated in Howick *et al.*, 2019 from *P. berghei* infections (see below and the new Figure 1B). Evaluating the effect of sequestration by comparing the profiles of *P. vivax* with those of *P. falciparum* is challenging since all available scRNA-seq datasets for *P. falciparum* are derived from *in vitro* cultures using different conditions and/or time points to obtain all asexual stages. Howick *et al.*, 2019 included 22 individual *P. falciparum* parasite cells obtained directly from clinical samples and those represented only male, female and early asexual parasites, presumably because of sequestration of mature asexual stages, but the very small number of *P. falciparum* cells analyzed limits further extrapolations.**

4. I feel the authors could make much more thorough use of available scRNA-seq data for blood-stages of Plasmodium. I understand the authors point in the introduction that prior studies have looked at cultured RBCs rather than cells harvested from an infected host. This is an important point, but in my view, that is all the more reason to compare the data more thoroughly. At present, the only comparison between P. vivax and P. falciparum in the current analysis is restricted to quantifying the proportion of the P. vivax transcriptome associated with genes that have or don't have a P. falciparum orthology. Why not also look at the relative stage specificity of these orthologs in existing scRNA-seq data? Ultimately, it is of course the authors' choice as to what they choose to include in their study. This is not a trivial undertaking and it may be beyond the scope of what the authors' wish to do here. I also am aware that nothing frustrates an author more than a reviewer commenting on a study they think the authors could have done, rather than the one they did do. That said, I think such an analysis would add to the impact of the study and is worth considering.

**The recent study by Howick *et al.*, 2019 provides a comprehensive comparison of the gene expression profiles across different *Plasmodium* species and we would therefore prefer to focus our analyses on our data and the specificities of *P. vivax* gene expression. We have, however, performed and included a new comparison with the scRNA-seq data obtained, using the same technology, from *P. berghei* blood stage infections (Howick *et al*., 2019). Overall, this analysis confirms our interpretation of the gene expression profiles obtained for *P. vivax* and suggests that the global regulation of gene expression is conserved between *P. vivax* and *P. berghei* (Figure B below and Figure 1B in the manuscript). Note however, that the results of this analysis probably accentuate similarities between species since we restricted the gene expression analyses to orthologous genes and did not consider species-specific genes. In addition, the overall similarity in profiles occults the fact that the proportion of mRNA molecules derived from genes with orthologs in the other species vary according to the stage of the parasites: while >80% of the mRNAs in trophozoites, early schizonts and female gametocytes derive from genes with a one-to-one orthologs in *P. berghei*, this number drops to less than 40% in late schizonts (see also Figure S19 in the revised manuscript). A detailed presentation of these results, as well as a more in-depth look at the gene expressed in male and female gametocytes, is now included in the revised manuscript.**

**Figure B. PCA showing the gene expression profiles of *P. vivax* (in grey) and *P. berghei* (in color) parasites based on the expression levels of 4,265 one-to-one orthologous genes. The color for the *P. berghei* parasites correspond to the annotations from Howick *et al.*, 2019: rings in red, trophozoites in green, schizonts in blue, male gametocytes in purple and female gametocytes in orange.**

Overall comments: This study is interesting and provides very valuable data that will be of broad interest to the field. I think identification of signals and markers of sexual-stage differentiation in P. vivax are important and again, will be of broad significance. However, the paper as written has some limitations in my view that must be addressed. Most significantly in the analytical stages in relation to the data normalization and differential gene expression analyses. These approaches are largely acceptable for bulk RNAseq data (with some caveats as noted above) but are not directly transferrable to scRNA-seq data. In my view, this has to be addressed before any further consideration of the manuscript can be made. I think the authors can make more of the comparative analyses with existing scRNAseq data for blood-stages of other Plasmodium species, but this is my subjective opinion and if the authors' choose not to expand the scope of their study, I won't stand in the way.

**We thank the reviewer for these suggestions and constructive comments that have significantly improved our manuscript.**

Reviewer #2:

Major comments:

In general, the data for this paper are highly valuable and impressive but the paper somewhat suffers from an underwhelming use of the data that should be rectified before publication.

This paper presents a bespoke analysis of P. vivax single cell data and would benefit from demonstrating that their approach is robust by comparing it to some of the more commonly used tools, for example at the very least presenting the CellRanger results. If the annotation of the genome is an issue and leads to poor results from CellRanger, then they could consider extending the annotated genes by a set amount from each start and stop (e.g., include 500bp up and downstream for each gene). It is unclear why they do not use popular and useful tools, and instead go about creating their own methods of analysis.

**We might have incorrectly conveyed the idea that our analysis pipeline was very different from CellRanger while it is heavily based on it and conceptually identical (we have now clarified this point on page 4). We initially spent a fair amount of time trying to get CellRanger to work on our data and would have preferred to use this approach directly. We eventually chose to write our own pipeline, recoding the different steps implemented in CellRanger to allow us to more easily modify and control every parameter. As the reviewer accurately notes, the gene annotation was one key issue driving this choice: as indicated in the manuscript, only 42-48% of the reads mapped within 500 bp of the annotated 3’-end of a gene. In addition, we wanted to be able to analyze the precise genomic location of the scRNA-seq signals and avoid collapsing signals that might represent different transcripts (as shown for example on Supplemental Figure S14). Arbitrarily extending the 3’-end of each gene, as suggested by the reviewer, does improve the number of reads assigned to genes but does not alleviate our other concerns, nor allows conducting all the analyses described in the manuscript. We were also concerned that the criteria used by CellRanger for discarding cells with too few reads (all cells with 1/10^th^ of the reads of the 99tile cells with the most expression) might fail to capture the range of transcriptional activity observed along the intraerythrocytic cycle (this concern turned out to be valid as Howick *et al.* 2019 had to manually “rescue” some cell populations that were systematically discarded by the CellRanger’s thresholding).**

The authors’ use of windows rather than genes should be tempered by providing the gene-based analysis first. It is somewhat concerning that the screenshots of IGV seem to show transcription primarily outside of gene boundaries. This may very well be UTRs and indeed, the patterns of transcription that are presented support the notion that windows approach works, but it would still be good to provide insight into differences in patterns or indeed loss of information when using only annotated gene boundaries. Some stats on genes with and without annotated UTRs would also be important, as if UTRs are annotated, then the windows approach becomes less robust.

**Because of the design of the 10X assay, which captures mRNAs with oligo-dTs and ligates the sequencing adapters after cDNA fragmentation, almost all scRNA-seq reads derive from the ~300 bp immediately preceding the polyA tail and therefore map to the 3’-UTRs (and not to the core of the gene). Given the incomplete annotation of the 3’-UTRs in *P. vivax* (none of the *P. vivax* P01 genes have 3’-UTR longer than 10 bp), it is not surprising that most reads map outside genes. Limiting our analyses to the current gene annotations would not only dramatically reduce the number of useful reads but could also collapse signals from different (unannotated) transcripts together and muddle the analyses. We did however summarize the bin data by genes and present these findings to facilitate the interpretation of the data (with a cautionary note on page 5).**

The authors state that they have observed “exquisite regulation of P. vivax transcription along the intraerythrocytic life cycle” but support this claim with a heatmap of a small subset of genes. A more global analysis of the transcriptional changes over developmental time in needed. Do you observe abrupt patterns of expression as seen in other single cell Plasmodium data sets (Reid et al 2018), and are the timing of expression patterns similar to other species (e.g. Poran et al 2017, Howick et al 2019)?

**We thank the reviewer for bringing this point to our attention. Reid *et al.*, 2018 describes three distinct clusters of genes based on their expression in asexual parasites and interpret this pattern as indicative of “abrupt changes” in transcription, contrasting with the continuous cascade of transcription and smooth transition observed by bulk-RNA seq (see e.g., Ho *et al*, 2016 or Bozdech *et al*, 2003). However, while their figure 4C seems to show distinctive clusters of genes, the PCA in Reid *et al* shows a continuous distribution of the asexual parasites and not discrete clusters as we would expect if there were “abrupt” changes in expression. The heatmap shown in their Figure 4 also seems to indicate that each gene is not either on or off but gradually increasing or decreasing in expression (though we would agree that this is quite subjective). The three clusters of genes described in Reid *et al.*, 2018 are different from what we observed in our data (e.g., our new Figures 2A, 3 and 4) and we wonder if this could be caused by an uneven distribution of the parasites along the intraerythrocytic cycle in the Reid study which could introduce some apparent clustering. We have included a comparison between our data and the recent *P. berghei* data generated by Howick *et al.* with the same technology as used in our study. This analysis highlights the similarities in transcriptional changes between these species along the intraerythrocytic cycle (see e.g., Figure 1B) and, we think, the conserved continuous changes in gene expression throughout the parasites’ development. We have also included a short discussion on this aspect on pages 13-14.**

The authors state that they have controlled for development by including pseudotime as a covariate. It’s important that they illustrate how this correction affects the results, as it seems to not capture how genes would be differentially expressed in a pseudotime dependent manner.

**The differential gene expression analyses were performed to study the effects of chloroquine and host monkey species on the gene expression of *P. vivax* parasites, not to evaluate which genes were differentially expressed according to the pseudotime. The pseudotime was used as a covariate in these analyses since the distributions of the parasites along their developmental trajectories might be slightly different in different samples (e.g., in *Saimiri* vs. *Aotus*) which could lead to spurious associations. Note that this approach allows detection of genes that are differentially expressed, upon chloroquine treatment or according to the host species, even if those genes are only expressed in some of the stages (see e.g., Supplemental Figure S20).**

The authors conclude that because the putative male population of cells doesn’t express genes associated with mature gametocytes that the known marker genes for males are not accurate. That these are definitely mature male gametocytes is unconvincing, and a more in-depth comparison to other published expression data sets in P. falciparum and P. berghei in needed including this recent biorxiv paper: van Biljon R, et al bioRxiv. 2019. p. 633222. doi:10.1101/633222.

**We are not sure we understand the reviewer’s comment. We identified this population of cells as male gametocytes because they express male gametocyte markers that were demonstrated in previous studies (e.g., MGET in Meerstein-Kessel *et al.*, 2019). This is now clearly indicated on page 10. Our revised manuscript also provides new information demonstrating that the profiles of both male and female *P. vivax* gametocytes align with those of *P. berghei* from the Howick *et al.*, 2019 study (Figure B and Figure 1B in the manuscript). As suggested, we have added a new table (Table S3) presenting genes highly expressed in *P. vivax* and *P. berghei* gametocytes, as well as whether these genes were detected in Yeoh *et al.*, 2017 and Obaldia *et al.*, 2018. These results are now discussed on pages 11-12.**

Throughout the paper the authors focus on individual genes or subsets of genes but don’t include any functional follow up or global analysis to support their conclusions. For example the authors look at expression of the MSPs and PIRs but discuss them in the context of all gene families. Is this pattern generalizable? Can the authors comment on function about the other gene families given their patterns of expression?

**We thank the reviewer for these suggestions and have implemented them. We chose to focus primarily on MSPs in our discussion as these represent one of the most studied multigene families in *P. vivax*. Other multigene families, such as PHIST and tryptophan-rich antigen, are less understood. Our revised manuscript briefly discusses the heterogeneity of expression of additional multigene families (on page 14) and provides new figures in support of our statements (Figures 4, S16-S18).**

DE assessments are not really using advantages of having single cell data at all, and presumably could have been done using bulk approaches with various purification methods to gather different stages. Consider fixing this. Also consider incorporating more of the power that single cell approaches give to examine things like variable gene expression, transcription factor coexpression, etc.

**We respectfully disagree with the reviewer on this point. Our take-home message is that, given our observation that the gene expression of most genes varies continuously during development, it will be very difficult to rigorously correct differential gene expression analyses generated from bulk RNA-seq: even a small difference in cell populations between samples could induce spurious results. This is especially true for species, such as *P. vivax*, for which tight synchronization or stage-specific “purification” is difficult. Analyses of scRNA-seq data using a statistical correction for the developmental stage of each cell circumvents this issue and can provide a robust assessment of differential expression. We agree that scRNA-seq is not a perfect solution as it will, for example, have limited power to detect differences at lowly expressed genes (such as transcription factors) due to its lower sensitivity. Our revision expands a discussion of the advantages and limitations of scRNA-seq on page 17.**

Minor comments

1. Many minor grammatical issues throughout.

**All hopefully fixed.**

2. The statement in the introduction “Unfortunately, the requirement for fresh and viable parasites has so far precluded extension of such studies to other Plasmodium species” should say instead something about “unculturable human species”. Technically, this is also inaccurate as P.malariae is present in the Howick et al bioRxiv paper.

**We changed the statement to “Unfortunately, the requirement for fresh and viable parasites has so far precluded extension of such studies to unculturable human *Plasmodium* species” as recommended by the reviewer. With regard to Howick *et al*., 2019, single-cell data were presented from only 22 *P. falciparum-* and 13 *P. malariae*-infected cells from patient samples. Since a typical 10X scRNA-seq experiment should provide information about 500-3,000 individual parasites, those results actually highlight the difficulties of conducting scRNA-seq analyses from *Plasmodium* parasites collected from *in vivo* infections.**

3. The authors state “We determined the number and viability of the purified cells” is stated in the methods, but no information is given on the results of the viability assays. Given the cells were enriched with MACS, the viability should be reported as well as when the viability assessments were made, given that three hours from sample to loading is a very long time. Were samples on ice this entire time?

**Information on the number and viability of purified cells is now included in Supplemental Table S1. Note that these results were obtained by BioRad TC20 Automated Cell Counter (as recommended by 10X Genomics) and rely on Trypan Blue exclusion test which assesses only whether the cell membrane integrity is maintained and does not differentiate infected from uninfected cells. The three-hour time span, from collection to loading on the controller, represents the maximum time. Typically, upon collection at the NIH, the blood samples were placed on ice, driven to UMB (~40 min) where they were processed on MACS columns at room temperature (~1h) before being loaded on the Chromium controller. We have now included these details on page 4.**

4. Not enough detail on loading for 10x and how this was accounted for given different parasitemias; not enough detail on why the runs varied so much in cells recovered.

**From each sample, we loaded an estimated 3,000 viable cells on the Chromium controller based on the cell counter estimates. We have not processed enough samples to understand the high variability noted by the reviewer but we speculate that this variability is at least partially due to the variation in parasitemia and the efficiency of the MACS column purification, which depends on the stage distribution (i.e., some samples may present more rings than mature/paramagnetic stages, therefore limiting MACS enrichment). As we process more samples, and test different enrichment methods we hope to get a better understanding of these parameters and obtain more consistent results from run to run.**

5. Was there any attempt to get rid of WBCs? Provide a basic analysis of the pure monkey cells to understand if these are RBCs or WBCs.

**The majority of WBCs were removed by the MACS column purification as they are not paramagnetic and therefore not retained. Based on the host gene expression profiles, more than 60% and 90% of the “pure monkey cells” are mature red blood cells in *Aotus* and *Saimiri*, respectively, and the majority of the remaining cells contain transcripts associated with platelets. We have included these results on page 9 and thank the reviewer for this suggestion.**

6. The number of lanes of HiSeq 4000 used should be reported.

**Most scRNA-seq libraries were sequenced in pools and sequenced across multiple lanes. The actual numbers vary depending on which libraries were prepared and sequenced together but the total number of paired-end reads generated is indicated on Tables 1 and S1.**

7. hisat2 is more typically referred to as HISAT2

**We have changed this throughout the manuscript. Thank you.**

8. Fig S7 should have the treatments/host labeled on it so that one can quickly identify which experiments are chloroquine treated, etc.

**Changed.**

9. Fig S10 purports to show that sexual and asexual parasites might use different UTRs for the same gene. From looking at the figure, it is also equally possible that those sexual parasites are just expressing the next gene along.

**In the specific case shown on (now) Fig S14, the orientation of the two genes (encoded on the same strand) excludes that they both contributed to the signals as suggested by the reviewer. However, we cannot rule out that one of the scRNA-seq signals derives from an unannotated transcript and that the two peaks correspond to two different genes rather than alternative UTR usage. This caveat is part of the rationale for analyzing bins rather than directly assigning scRNA-seq peaks to annotated genes. We have added text to make this point clear on page 5.**

10. “Most P. vivax genes remain unannotated.”, would be perhaps better stated as “..remain incompletely annotated”. With >6000 annotated genes in the P. vivax genome, it seems unlikely that there remain >6000 yet to be annotated.

**We apologize for the confusion. Our intention was to point out that i) the annotation of *P. vivax* genes is incomplete, especially regarding the untranslated regions (UTRs) which are essentially lacking from the PlasmoDB annotation, and ii) that many genes are not functionally annotated (i.e., they are often labelled as “conserved protein of unknown function”). We have clarified these points on pages 14 and 15.**

11. Fig 4 also needs some kind of normalizer that shows the total number of genes identified along pseudotime rather than just the proportion that have a P. falciparum ortholog.

**Figure 4 (now Figure 5) does not show the proportion of expressed genes with a *P. falciparum* ortholog but the proportion of transcripts (i.e., mRNA molecules) that are derived from *P. vivax* genes that have one or more *P. falciparum* orthologs. This number is not dependent on the number of genes expressed. We have rewritten the legend of the figure for clarification.**

12. The authors state “To statistically determine whether specific transcripts were differentially regulated upon chloroquine treatment, we compared the gene expression profiles of the parasites from the same Aotus infection before and 20-hour post- treatment, adjusting for differences in developmental stages” needs further elaboration on how you adjust for differences in developmental stages.

**This is now described in more details in the Methods section on page 7.**

13. No comment is made on the drug resistance status of the strains used; do any of  them carry mutations associated with drug resistance?

**We now provide this information on page 4.**

14. When discussing the different host species, it would be beneficial to remind the reader an estimate of how many rounds of re-invasion (length of time post inoculation) the parasites have been through.

**The length of time post inoculation is now included in Table 1 and Supplemental Figure S1. Thank you for the suggestion.**

15. When referring to reference 57, which seems to investigate only Saimiri monkeys, it would be useful to discuss the locations from where the various P. vivax strains under discussion originate from. Indeed, this information appears to be completely missing from the methods/materials and I can only find strain names in table 1, with no explanation on what these are or where they originate from.

**Gunalan *et al.,* 2019 performed bulk RNA-seq analysis of *P. vivax* Salvador-I in both *Saimiri* and *Aotus* monkeys. As suggested, we now provide the geographic origin of each line used in this study on page 4.**

16. Figure S4 is meant to show that the data do not change based on the QC cut off on reads per cell. However, it only demonstrated that the general shape of the PCA is the same (and is really hard to see the points in grey). It would be helpful to quantify how each developmental stage or cluster is represented depending on the cut off used.

**A new figure (Supplemental Figure S5) has been provided. Thank you for this suggestion.**

17. Figure 3 illustrates non-overlapping expression of 4 AP2 transcription factors. How many of the ~27 AP2s were detected and how common was this stage-specific expression?

**To answer this question, we provide a new figure (Supplemental Figure S13) showing the expression of all 28 putative AP2 domain transcription factors annotated in the *P. vivax* P01 genome. As noted in our responses to Reviewer #3 (see below), we have extensively revised the discussion of AP2 gene expression and moved much of the information to supplementary files.**

18. Figure 5: label axes

**Done.**

19. The authors state “Finally, we discarded all individual parasite transcriptomes containing less than 5,000 unique reads mapped to the P. vivax genome as well as those containing more than 75,000 unique reads (Table S1)” & “Figure S4. Principal component analysis of the 13,503 individual parasite transcriptomes each containing more than 1,000 unique reads. Each dot represents a single parasite transcriptome and is displayed according to its gene expression profile along the first three principal components. Note that the figure is virtually identical to the PCA generated using the 9,215 transcriptomes with more than 5,000 unique reads (Figure 1).”....These cut offs seem high - which cells do you lose with this filtering? Is it cell type biased?

**Lowering the cutoff from 5,000 to 1,000 unique reads did not change the overall distribution of cells along the intraerythrocytic cycle, apart from a small increase in the proportion of late asexual stages. We have included a new figure (Supplemental Fig. S5) to show this. The general shape of the PCA plot and the overall conclusions remained the same with both cutoffs.**

20. The authors state “The analyses described above did not reveal any major effect of chloroquine (or of the host species) on the overall patterns of gene expression: we observed all parasite developmental stages in most samples regardless of the treatment or the host, and the transcriptomes of treated parasites or parasites from different hosts overlapped apparently perfectly (Table 1 and Figure S13).” and from methods: “We conducted these paired analyses using EdgeR 36, independently for female gametocytes and asexual parasites, and further subdivided the latter into four crude developmental categories along the intraerythrocytic cycle”. It is important to go into more detail of how you divided asexuals, and a single-cell specific differential expression tool should be used to account for dropout.

**We have extensively modified the differential gene expression analysis section according to the suggestions of the Reviewer #1. The requested experimental details are provided in the Methods section of the manuscript.**

Reviewer #3:

Overview:

The authors should be commended for their heroic undertaking to determine several Plasmodium vivax transcriptomes from in vivo infections using single cell RNA-seq. These are the first example of such data which builds on data the group has previously generated by replacing bulk transcriptomics from patient isolates with single cell RNA-sequencing data generated from the infected reticulocytes of splenectomized monkeys. Using dimensional reduction based analysis of the data, the authors are able to characterize the probable sequence of gene expression events throughout P. vivax asexual development and to some extent during male and female gametocytogenesis. Despite the potential richness of the datasets acquired in this study, the manuscript fails to deeply analyze the data, providing a very broad over-view of the P. vivax in vivo transcriptome that ultimately fails to deliver on new biological insights, rather confirming previous studies. The manuscript also appears to have been put together hastily and therefore is difficult to read at times and repetitive in other sections, lacking significant editing prior to submission.

General Comments:

It is difficult to understand from the manuscript what new information was learned relative to The transcriptome of Plasmodium vivax reveals divergence and diversity of transcriptional regulation in malaria parasites (2008 PNAS). Is the novelty in the unique markers that cannot be picked up by bulk RNA-seq?

**We consider that the scRNA-seq results presented here complement the seminal study by Bozdech *et al.*, 2008 and provide both new insights and a solid foundation for future research. The 2008 study relied on a handful of patient samples preselected for the “synchronous *P. vivax* population at ring stage” and cultured *ex vivo* prior to gene expression characterization. The collection of RNAs at discrete intervals along the intraerythrocytic development provided valuable profiles of gene expression for the different stages, but did not allow to interrogate whether the differences in gene expression occurred abruptly or continuously. In addition, given the difficulty to culture *P. vivax ex vivo,* it is not entirely clear how “synchronized” the cultures remained over time, which could be problematic since contamination by even minute amount of another stage could have a dramatic impact of the overall gene expression due to the extensive variation in transcriptional activity (see below). Another possible limitation of the 2008 study is the possible effect of the *ex vivo* culture on the parasite gene expression that was highlighted (for another *Plasmodium* species) by the authors in a later publication (Lapp *et al*., 2015). The scRNA-seq study described here, while not free of limitations, corroborates most findings from Bozdech *et al.*, 2008 while providing a more granular perspective on the changes in gene expression. Our study also provides new insights on the gene expression profiles of male compared to female gametocytes, and suggest novel markers of sexual stage development that have been challenging to obtain for *P. vivax*.**

Furthermore, since in the previous study the authors could culture the parasites ex-vivo and therefore determine the morphology of the parasite unambiguously, the authors should compare their data to this study to see if the highest correlations exist at the expected stage (i.e. the trophozoite and schizont datasets correlate highly, whereas the ring data is missing as they speculate is the case in their sc-RNA-seq asexual transcriptome?).

**Previous gene expression deconvolution analyses seem to suggest that *ex vivo P. vivax* parasites lose their synchronicity over time and that, at the latter time points, the cultures contain less tight populations of parasites than expected. To address this possible issue and concerns about the impact of the *ex vivo* culture on the gene expression profiles, we have compared the gene expression profiles from our experiments with those of *P. berghei* parasites obtained in a similar manner (as suggested by the reviewers and the editor). This comparison is now presented on page 10 and provides further evidence for our previous interpretation of the gene expression profiles and the loss of rings (see Figure B above and Figure 1B in the manuscript).**

Furthermore, how does the proportion of genes detected in this sc-RNA-seq study compare to bulk studies? At what threshold do you no longer detect genes that previous datasets have determined to be expressed but at a low abundance?

**One limitation of the 10X scRNA-seq platform is that it yields a fairly “shallow” description of the gene expression profiles and only provides robust and reliable information on highly expressed genes. We included a new table (Table S2) to highlight this limitation. On the other hand, while yielding a deeper characterization of the transcriptome, bulk RNA-seq provides a weighted average of the gene expression of all stages present in the infection but, due to their much higher transcriptional activity, is highly biased towards trophozoites genes (see Kim *et al.,* 2019 and references therein). Thus, while bulk RNA-seq offers a deeper characterization of trophozoite transcripts, the amount of specific information it provides on other stages is not clear, as this depends on the relative proportion of the stages in the infection and their relative transcriptional activity. For studies in which synchronized parasites can be obtained in large numbers (e.g., from *P. falciparum* cultures) stage-specific RNA-seq can provide robust information on low-level transcripts (though artifacts can still arise from imperfect synchronization and contamination from parasites from other stages). Interestingly, we observed that only a few of the male gametocyte specific genes identified in the scRNA-seq data were detected in our previously published Cambodian patient RNA-seq data (Supplemental Figure S11), suggesting that for some stages the scRNA-seq data might actually be more sensitive than bulk-RNA seq.**

Can the authors graph their phaseogram against that of the orthologous genes in a better characterized Plasmodium spp. (I.e. falciparum or berghei) to highlight the differences and similarities across malaria parasites?

**We thank the reviewer for this suggestion and have added new figures comparing the gene expression in *P. vivax* and *P. berghei* (Figure 1B and Supplemental Figure S8).**

Can the authors use the high quality paired end reads they have generated to assemble a putative P. vivax transcriptome de novo? It would be both interesting and of great use to the field to have a more reliable P. vivax transcriptome to map NGS sequencing data to, or conversely to provide higher confidence in the currently predicted gene models.

**We absolutely agree with the utility of such a resource. Unfortunately, the 10X scRNA-seq reads derived almost exclusively from the last 300 bp of each transcript precluding *de novo* assembly of the transcripts (see the response to Reviewer #2 for more details). We are in the process of testing different protocols to combine single cell RNA-seq with long read sequencing (e.g., Pacbio) but this work is still very preliminary and outside the scope of the present study.**

There is a missed opportunity in the authors’ exploration of the expression of AP2 domain proteins throughout asexual and sexual development. 12/27 of the AP2 domain proteins predicted in P. vivax have been genetically characterized to some extent in other Plasmodium species. How do their temporal expression patterns match their genetic roles? We feel that the PfAP2-I orthologue may be of particular interest due to its role in activating transcription of genes needed for red blood cell invasion in Pf. Although the essential AP2 domain (D3) of PFAP2-I is 100% conserved between Pv and Pf (implying that it will bind a similar DNA motif) the Pv invasion pathway is presented as being different and presumably involves the transcriptional activation of different genes, as the authors have pointed out.

**We attempted to conduct additional analyses of the AP2 domain proteins as suggested by this reviewer and encouraged by the editor (see also below). Unfortunately, the current data are very limiting because of the low level of expression of many AP2 genes. Considering this limitation and the extensive information on AP2 gene regulation available from previous studies, we would like to move most of the AP2 section to supplemental information and, thus, make room for the comparisons with *P. berghei*. We are working to obtain greater depths of scRNA-seq data, which could help to tackle the above questions and pursue the analyses suggested by the editor.**

In keeping with the above content, can you use the existing Pv gene models to predict DNA cis-regulatory motifs based on the temporal profile of mRNA abundance? This would provide a very convincing argument that the precisely regulated timing of transcription factor expression is relevant for controlling the rest of the transcriptome, as the authors have suggested.

**We tried to identify clusters of co-expressed genes (using a modified version of WGCNA) to then search for enriched motifs in the 5’ region of each gene but failed to obtain convincing modules (probably due to the high number of drop-offs). At this point, we feel that our current data are not sufficient to address this question.**

Specific Comments:

Page 2 line 18- In the following citation, ex vivo cultured P. falciparum isolates were used to address some of the shortcomings mentioned, specifically the fact that cultured parasites lose the ability to express genes that are important for host interaction.

Blythe, J. E., Xue, Y. Y., Kuss, C., Bozdech, Z., Holder, A. A., Marsh, K., … Preiser, P. R. (2008). Plasmodium falciparum STEVOR proteins are highly expressed in patient isolates and located in the surface membranes of infected red blood cells and the apical tips of merozoites. Infection and Immunity

**Thank you for this suggestion. We have now included this reference in our discussion.**

Page 2 line 17- Was there any way for the authors to look at their parasites microscopically before generating the material for an RNA-sequencing library (as in reference 29)? How robust is the pseudotime data reduction i.e. can the age and life stage of parasites analyzed in this manuscript be said to be known with absolute certainty?

**We prepared blood smears from each sample and counted an estimated 10,000 red blood cells prior to processing through MACS columns. We agree that it would have been better to also have a second smear after MACS enrichment, but we did not have enough sample from the column. We have now included in Table 1 the stage composition determined by blood smear from each sample (prior to MACS enrichment).**

**Regarding the calculation of the pseudotime, we believe that this metric captures most of the variation occurring along the developmental cycle. The absolute value of this estimate is probably not very informative (as it depends on changes in gene expression and on the distribution of the parasites along the intraerythrocytic cycle) but the ranking reflects morphological changes in the parasites, appears robust with regards to the data cutoffs and normalization (see above), and is correlated with the gene expression variation observed in *P. berghei*.**

Page 4 line 21- how does mapping the artificially trimmed 75bp vs 101 bp reads compare? If you use the longer reads do you increase your information about gene structures?

**We typically use 50 bp or 75 bp reads in our RNA-seq experiments and have not observed any significant mapping problem (i.e., the vast majority of the reads map unambiguously to a single location in the *P. vivax* or host genome). Increasing the read length would not really help to better understand gene structure as the cDNA molecules sequenced are still restricted to the last 300 bp of each transcript. Longer reads would be informative only if combined with a different fragmentation process than the one implemented in the 10X 3’-end scRNA-seq protocol (and we are currently testing different approaches to do so).**

Page 5 sequence analysis- Is it normal with single cell transcriptome data to normalize read depth across bins as you have described? Is there a significant difference in the sequencing depth originating from different locations on the transcriptome? Can you provide a sc-RNA seq citation that analyzes the reads in a similar way?

**There may have been some misunderstanding of our description of the normalization process. We did not normalize read depth across bins but normalized the read counts per parasite to account for the overall number of mapped reads obtained from each droplet. This is a standard and necessary normalization of scRNA-seq and RNA-seq (sometimes referred to as scaling normalization or library size normalization). We have edited this section on page 5 to clarify our approach. For the principal component analyses, we normalized (scaled) the variance of each bin to avoid disproportional influence of highly express genes. We have clarified this point on page 6.**

Can the authors provide an updated putative P. vivax transcriptome based on the gene structures (splice junctions, UTR coverage, etc.) detected in their data? Although that the goal of this work was to quantify transcripts using the 3’ end reads, the dataset should also impart some new insights into the transcriptome?

**Since the vast majority of the 10X scRNA-seq reads are, by design, derived from the last 300 bp immediately preceding the polyA tail of each transcript, very few reads overlap a splice junction and these data provide little information pertaining to gene structure.**

Page 7- The Results section describing “detailed transcription information” contains numerous arbitrary facts concerning the number of total reads and fraction pertaining to host versus P. vivax. In the end, this serves to confuse the reader more than providing clarity and diminishing the significance of the number of reads attained in this study.  This section also describes a rational for inclusion of certain transcriptomes and not others which comes across as arbitrary without any statistical metric for inclusion/exclusion.

**We have edited this section for clarification. We agree with the reviewer that some of these cutoffs are arbitrary (as are those used by CellRanger or other scRNA-seq packages) and we attempted to justify the choice of our cutoffs in the manuscript (e.g., to maintain adequate read counts across cells used in the analysis). We also included results using alternative cutoffs (e.g., Supplemental Figures S4 and S5, and the response to Reviewer #2) to show the robustness of this approach. More details are now provided on page 9.**

Page 7 line 25-29- Isn’t the information provided here contradictory? How can the spread of reads mapped to Pv be 40 to 80% but the majority of datasets are either all host or all Plasmodium?

**While 40-80% of the reads generated from each blood sample map to the *P. vivax* genome, most cells in each sample contain either monkey or *P. vivax* reads. The overall 40-80% is due to i) the presence of more cells containing *P. vivax* reads than cells containing monkey reads and ii) a higher average number of reads in *P. vivax* cells than in monkey reads. For example, if we generated 1,000 cells from one blood sample and obtained 800 *P. vivax* transcriptomes and 200 transcriptomes from monkey blood cells, the proportion of reads mapped to *P. vivax* could be 80% (assuming the same number of reads in *P. vivax* and monkey cells) while all transcriptomes would be exclusively made of *P. vivax* or monkey reads. See Supplemental Figure S3 for details.**

Page 8 line 16- Please state how many genes 19,282 unique sequences corresponds to in your dataset.

**Determining the number of genes expressed per cell, or overall per sample, is not straight-forward. Most published scRNA-seq studies considered that one read is sufficient to consider a given gene expressed but this approach likely has a high false positive rate and overestimates the number of genes expressed. To balance these figures (presented in Supplemental Table S2), we also included a more conservative estimate based on the number of genes accounting, in each cell, for more than 1% of the total mRNA molecules (e.g., more than 50 reads in a cell with 5,000 reads). This discussion is included in the text (page 9) and the numbers provided in the new Supplemental Table S2 together with the corresponding numbers calculated for other previous *Plasmodium* scRNA-seq studies.**

Page 9 line 10- Is there a citation for the group of male gametocyte genes in P. vivax? Otherwise how were these predicted?

**We thank the reviewer for bringing this omission to our attention. We have added the references for both male and female gametocyte markers used.**

Page 9 line 20- How deep of sequencing do you need to detect SNPs with high confidence? Does this data consistently reach that threshold?

**For the analyses presented in this manuscript, we only used SNPs to authenticate the monkey-adapted strains used for the infections and performed the genotyping at the sample level (i.e., not at the single cell level which would require a different approach with different cutoffs). As indicated in the Methods section on page 8, we examined positions sequenced by >20 reads in a given sample which is the cutoff we typically used in our genomic studies (and is more conservative than the cutoff used by many groups for similar analyses). This cutoff provides a very high confidence on our genotyping calls as demonstrated by the independent genotyping of the same strain: out of 2,279,246 positions examined, we observed 0-1 spurious differences between the genotype of the strains analyzed in the same monkey host four days apart (before and after CQ exposure), and between infections of the two *Saimiri* monkeys with the same NIH-1993 strain (see also Supplemental Table S6).**

Page 9 – How were the genes selected for inclusion in Figure 2?

**These genes were selected to illustrate the findings discussed in the manuscript. Along with our modifications of the text and presentation, we provide a new figure (Figure 2A) showing the transcriptional variation of the *P. vivax* genes most expressed along the intraerythrocytic cycle (calculated by binning the parasites into 10 categories according to their pseudotimes). Other figures (2B, 4A and 4B) and supplemental figures display data for different gene families or markers.**

Page 10 line 20- Is it possible that the sc-RNA seq method does not adequately detect low abundance transcripts, and this is why previously theorized markers were not detected? What fraction of predicted P. vivax transcripts were detected relative to the previous bulk RNA-seq study in reference 18?

**Please see our response to the relative advantages of each method above. It is indeed possible that previously proposed markers were not detected due to the lower sensitivity of scRNA-seq. However, we only discussed genes that were successfully detected at least in some parasites. The comparison with the bulk RNA-seq is difficult as the proportion of transcripts detected will depend on the stage in which they are expressed: as discussed above, bulk RNA-seq efficiently characterizes the transcripts of trophozoites but has lower power to detect genes expressed in less abundant stages in the infection (e.g., gametocytes) as well as in stages that, proportionally, generate less mRNA than trophozoites (see also the response to Reviewer #2 for a detailed discussion).**

Page 10 line 31- P. vivax has direct orthologs to ap2-g2 and ap2-g3, which are thought to be important for gametocyte development in all Plasmodium species where they have been genetically interrogated. Are their transcripts detected in either gametocyte population? It would be useful to provide a comprehensive list of all predicted gametocyte-related genes (many are now available in the literature) and list their P. vivax orthologues to better define new versus previously suspected sexual stage genes emerging from this study.

**The list of transcripts expressed in gametocytes based on our scRNA-seq data is presented in Supplemental Table S3 (together with the specificity and a comparison to *P. berghei* data).**

Page 11- I believe that AP2 domain proteins should be written as AP2 (or ApiAP2), not AP-2
Many of the genetically characterized ApiAP2 proteins in Pb and Pf have orthologues in Pv and the important invasion transcription factor AP2-I has 1:1 conservation in its 3rd AP2 domain, implying that it probably binds a similar DNA sequence. Can you find any temporal conservation of potential 5' cis regulatory DNA elements in your data that implicate any of the AP2 domain proteins in the observed expression cascade? This may be especially interesting for AP2s involved in invasion (such as AP2-I) because or the differences in invasion mechanism between Pf and Pv.

Bozdech et al. previously proposed a cascade of regulation through AP2 proteins in their in vitro P. vivax transcriptome paper (2008).

**We have added this citation and references to additional studies on the successive expression of AP2 genes in the text (page 13). Based on these previously published data, we felt that our findings were not as novel nor unexpected as we initially thought and significantly shortened the discussion of the AP2 results. We have renamed the genes as requested by the reviewer. We attempted to look for TFBS motifs in genes co-expressed with specific AP2 genes but our current data are not sufficient for robust analyses (see also the response to Reviewer #2 for details).**

Page 12- The authors state that the current gene annotations are rather poor for the P. vivax genome? Is this actually correct? What is the current number of gaps in this genome and by homology and synteny alone, can’t the counter argument be made that the genome is in quite good shape?

**The *P. vivax* genome sequence is indeed quite good as highlighted in many whole genome sequencing studies including from our lab. However, the gene annotations are still incomplete, both physically, with many UTRs not being annotated, and functionally, with many genes having no clear annotation (i.e., being labeled as “conserved protein” or “hypothetical protein”). On page 15, we specifically refer to the lack of functional annotations and have now clarified this point.**

Page 12-13- Can you please provide a biological rationale why there would be a dramatic variability in the number (up to 63.4%) of genes expressed that are absent in P. falciparum during invasion? I can appreciate that invasion would be different for Pv, but why should it be so different across the measured cells? Some don’t appear to vary much at all, while others vary a lot.

**We thank the reviewer for noticing this aspect of the data: while the vast majority of the mature asexual cells showed a significant drop in the proportion of transcripts derived from genes with one (or more) *P. falciparum* orthologs, this is indeed not true for all of them (see also Figure C). We are not entirely sure why this is. Note that this drop in orthologous transcripts in schizonts is also observed in the comparison of *P. vivax* with *P. berghei* (Supplemental Figure S19).**

**Figure C. PCA of the *P. vivax* transcriptomes colored according to the proportion of transcripts derived from genes with orthologs in *P. berghei* (in red scale).**

Page 13 line 10- what does “apparently perfectly” mean in this context? Is the correlation between the datasets really 100%? There is no statistical basis for this statement.

**We agree with the reviewer and have removed this statement.**

Page14- if the authors optimized their isolation to find reticulocytes with mature parasites could the undetected subpopulations (related to the Duffy Binding Protein) just not be enriched in the purification process, as the authors already suggested for rings?

**The isolation of infected red blood cells utilizes the paramagnetic property of hemozoin and selects for cells in which the parasites have digested hemoglobin over time. While this approach fails to efficiently capture immature parasites (that did not have enough time to produce sufficient amount of paramagnetic hemozoin), it is unlikely to differentially capture parasites using different invasion pathway.**

Figure 1- please add the gene names to the Gene ID (e.g. AMA1, Pvs25, etc…).

**Changed**

Figure 4- please clarify why there are different scales for each of the figures?

**This figure has been replaced.**

Miscellaneous-Pv is known to react differently to chloroquine than falciparum. Is there any reason to expect a transcriptional response that can be explained by falciparum biology?

**The original observation regarding a different mechanism of chloroquine resistance in *P. vivax* in relation to *P. falciparum* is based on the fact that distinct from *pfcrt*, no coding mutation in *pvcrt* is associated with chloroquine resistance phenotype (Nomura et al, 2001). While this has been confirmed by several studies and continues to be the case, further investigations using heterologous expression systems suggest *pvcrt* has a native capacity to transport chloroquine (e.g., Sa *et al.*, 2006; Baro et al., 2011; Hassett et al., 2017), and more recently a genetic cross has linked *pvcrt* expression to chloroquine response (Sa *et al*., 2019).**

Academic Editor's:

The work uses excellent cutting-edge technology to report several novel findings and several that confirm what was already known from bulk RNAseq or scRNAseq in other species

The expression of Pv specific genes primarily in late stage schizonts for invasion is an important and novel finding.

Interestingly the authors show that there appears to be a deletion in monkey passaged paras and that an msp3 is differentially expressed between monkey spp, this evidence of species differences could be informative for factors specific for human disease.

The lack of co-variation between female and male gam genes is interesting, ie the ratios are diff between patients. I find the observation sound although it is confirmatory of what the authors previously published in their bulk rnaseq data. However, I found the terminology used here confusing

“the terminal phases of male and female gametocytogeneses are independently regulated in P. vivax”

Actually they seem to be dependently regulated, so that the probability of one cell being female affects another cell in the same host hence the disparity in sex ratios between hosts, if they were truly independent wouldn’t they trend to 50/50 or have the same unequal ratio across all hosts?

**We thank the editor for this comment and have modified this sentence accordingly.**

H4 expression increased in cq treated paras, this is interesting in light of the known DNA intercalating properties of Cq but a lot of work over a long time has convincingly shown that the Cq kills via interfering somehow with haemoglobin digestion/hemozoin formation in the digestive vacuole so the authors need fairly strong evidence to propose resistance via increased histone expression, I think this section should be toned down.

**We have modified this section based on the editor’s comment and the suggestions of the Reviewer #1.**

Interestingly the alternative histone H3.3 is upregulated (Table S3), H4 might be upregulated because they are deposited as a dimer together post replicatively. H3.3 is involved in gene regulation in many species and in telomeric silencing but in P falciparum it has some dynamic association with promoters but is primarily constitutively associated with subtelomeric repeats and coding sequence (Fraschka 2016 SciReports). It is implicated in male gamete development in mammals so could be somehow associated with gam development, this hasn’t been investigated. Increased H3.3-H4 incorporation could be a stress response to Cq.

**We have modified this section and now present a more detailed analysis of histone expression on page 12.**

Two substantial aspects of the work were the ap2 lifecycle associations and the gender specific genesets. However I did not get a feel from the MS how different the vivax data were to already published datasets from other species. I think this needs to be more clearly discussed in light of the author’s own discussion of the need for P vivax studies due to its unique biology.

**We have modified the manuscript to emphasize the differences between *P. vivax* and other *Plasmodium* parasites and highlight how this work can improve our understanding of *P. vivax* biology (e.g., the differences in expression among members of multigene families).**

The expression of specific ap2 at diff stages suggesting they specifically regulate progression through the lifecycle. However the bulk culture RNAseq of P. berghei, (which is more closely related to P vivax than is P falciparum, although still quite distant) has already revealed the phased expression of sets of Api AP2 transcription factors (Modrzynska et al 2016 Cell Host Microbe) and importantly 12 of 26 ApiAP2 have been KOd/KDd and their role in stage specific development confirmed by functional evidence of lethality in various stages in this and other studies. The principle was thus established without the scRNAseq although not at the same resolution. The authors should discuss their findings in light of these other studies, are there results congruent with the other studies in other species?

**We thank the editor for these precisions. Based on this comment and other reviewers’ suggestions, we have significantly toned down this section and now describe more briefly our findings in the context of these previous studies on page 13. Also, please see our responses on AP2 expression in the general comments section above.**

“We next searched the scRNA-seq data to identify transcripts highly expressed in male and/or female gametocytes and with little expression in other stages to generate a comprehensive list of putative biomarkers for male and female gametocytes (Table S2).”

Similar analyses have been conducted using scRNAseq in P. falciparum and P berghei (Reid et al 2018 elife). Also bulk RNAseq of male and female gams in P berghei (Yeoh BMC genomics 2017) and P. falciparum (lasonder 2016 NAR). It would be useful to know how much of these genesets intersected with the current study and how much is unique to P vivax, also the extent to which scRNAseq identified novel sex specific genes compared to the earlier bulk RNAseq experiments. The findings of these previous study should be discussed by the authors.

**We have now included a new analysis comparing the genes expressed in male and female gametocytes in *P. vivax* and *P. berghei* (Table S3) and discuss our results in the context of the studies highlighted by the editor.**

Minor comments

the PCA plot in fig 1 is color coded by genes, the function of which is used as a surrogate of lifecycle stage but the function of the genes and their lifecycle association is not explicitly stated in the fig, its legend or the text.

**We have changed the manuscript accordingly and added a new figure (Figure 1B).**

Page 10 mature gametocytes (Figure S8), suggesting that these genes might be expressed only in female mature gametocytes”

This is not apparent to me from fig s8, it looks like the proportion of mature gam genes is as high as immature gam genes in males and the ratio of immature to mature is higher in males than females. The actual levels of expression of the mature gam genes in males does not seem to be presented in fig s8 at all.

**We have modified our statement on page 11.**

Page 14 dbp2 nomenclature should be clarified in the text, its called ebp2 in the fig legend and ebp in the fig

**Changed to DBP2.**

Table S3 colour coding re significance seems odd, the numbers are ranks for the genes significance of fold change but this is not informative to me, they may be highly ranked and still non-significant, the colour coding isn’t explained, I assume they meet some significance threshold?

**These previous analyses of differential gene expression have now been replaced by those suggested by Reviewer #1 and these tables have been modified. We describe the significance thresholds used in these analyses on page 7.**
