# Supplementary material for: Single-cell transcription analysis of Plasmodium vivax blood-stage parasites identifies stage- and species-specific profiles of expression
Source: PLoS Biol. 2020 May 4;18(5):e3000711. doi: 10.1371/journal.pbio.3000711 (PMC7224573; doi:10.1371/journal.pbio.3000711)
Supplement: Attachment — Submitted filename: SaetalRev2.docx [file pbio.3000711.s051.docx]

The authors have addressed the majority of comments well, but some important areas still require further clarification and possibly work. We have pasted the original reviewer comments and author responses that we think require further consideration or work below. Our additional comments are in red.

Reviewer 2

This paper presents a bespoke analysis of P. vivax single cell data and would benefit from demonstrating that their approach is robust by comparing it to some of the more commonly used tools, for example at the very least presenting the CellRanger results. If the annotation of the genome is an issue and leads to poor results from CellRanger, then they could consider extending the annotated genes by a set amount from each start and stop (e.g., include 500bp up and downstream for each gene). It is unclear why they do not use popular and useful tools, and instead go about creating their own methods of analysis.

**We might have incorrectly conveyed the idea that our analysis pipeline was very different from CellRanger while it is heavily based on it and conceptually identical (we have now clarified this point on page 4). We initially spent a fair amount of time trying to get CellRanger to work on our data and would have preferred to use this approach directly. We eventually chose to write our own pipeline, recoding the different steps implemented in CellRanger to allow us to more easily modify and control every parameter. As the reviewer accurately notes, the gene annotation was one key issue driving this choice: as indicated in the manuscript, only 42-48% of the reads mapped within 500 bp of the annotated 3’-end of a gene. In addition, we wanted to be able to analyze the precise genomic location of the scRNA-seq signals and avoid collapsing signals that might represent different transcripts (as shown for example on Supplemental Figure S14). Arbitrarily extending the 3’-end of each gene, as suggested by the reviewer, does improve the number of reads assigned to genes but does not alleviate our other concerns, nor allows conducting all the analyses described in the manuscript. We were also concerned that the criteria used by CellRanger for discarding cells with too few reads (all cells with 1/10th of the reads of the 99tile cells with the most expression) might fail to capture the range of transcriptional activity observed along the intraerythrocytic cycle (this concern turned out to be valid as Howick et al. 2019 had to manually “rescue” some cell populations that were systematically discarded by the CellRanger’s thresholding).**

The improved method sections provides clarity on why this bespoke analysis was performed; however, it would still increase the impact of the paper if there was a more in depth comparison between the CellRanger output and the window based count table. How much of the biology that is discussed in the manuscript is actually lost by using CellRanger? Should all Plasmodium RNA-seq studies on P. vivax use this new method from now on? The authors should provide at least one quantitative comparison of results using their bespoke method and CellRanger with UTRs extended in the GTF by a reasonable amount. This can be supplementary, and their main text can stay as it is, but it is important to do this.

The authors’ use of windows rather than genes should be tempered by providing the gene-based analysis first. It is somewhat concerning that the screenshots of IGV seem to show transcription primarily outside of gene boundaries. This may very well be UTRs and indeed, the patterns of transcription that are presented support the notion that windows approach works, but it would still be good to provide insight into differences in patterns or indeed loss of information when using only annotated gene boundaries. Some stats on genes with and without annotated UTRs would also be important, as if UTRs are annotated, then the windows approach becomes less robust.

**Because of the design of the 10X assay, which captures mRNAs with oligo-dTs and ligates the sequencing adapters after cDNA fragmentation, almost all scRNA-seq reads derive from the ~300 bp immediately preceding the polyA tail and therefore map to the 3’-UTRs (and not to the core of the gene). Given the incomplete annotation of the 3’-UTRs in P. vivax (none of the P. vivax P01 genes have 3’-UTR longer than 10 bp), it is not surprising that most reads map outside genes. Limiting our analyses to the current gene annotations would not only dramatically reduce the number of useful reads but could also collapse signals from different (unannotated) transcripts together and muddle the analyses. We did however summarize the bin data by genes and present these findings to facilitate the interpretation of the data (with a cautionary note on page 5).**

The new Figure S2 appears to indicate that >40% of observed transcription is more than 2.5kb away from an annotated gene. Is this true? It would be important to further discuss this in the manuscript if so, and draw comparisons with results from bulk RNAseq. Additionally, for the remaining windows that are within 1kb of a gene, it is important to do a more clear-cut gene-for-gene comparison to the other species scRNAseq data out there, as noted in the next section’s comment.

 A better way to display information currently contained within S2 would be a conventional coverage plot across all genes including multiple windows beyond the stop codon. If the authors think the unannotated transcripts that are more than 500 bp from an annotated gene are driving the biology in their data, more work needs to be done on when and where they are expressed. It would be reassuring to see that the reads that map to these new 3’ UTR regions in Pv map to 3’ UTRs in orthologous genes.

The authors state that they have observed “exquisite regulation of P. vivax transcription along the intraerythrocytic life cycle” but support this claim with a heatmap of a small subset of genes. A more global analysis of the transcriptional changes over developmental time in needed. Do you observe abrupt patterns of expression as seen in other single cell Plasmodium data sets (Reid et al 2018), and are the timing of expression patterns similar to other species (e.g. Poran et al 2017, Howick et al 2019)?

**We thank the reviewer for bringing this point to our attention. Reid et al., 2018 describes three distinct clusters of genes based on their expression in asexual parasites and interpret this pattern as indicative of “abrupt changes” in transcription, contrasting with the continuous cascade of transcription and smooth transition observed by bulk-RNA seq (see e.g., Ho et al, 2016 or Bozdech et al, 2003). However, while their figure 4C seems to show distinctive clusters of genes, the PCA in Reid et al shows a continuous distribution of the asexual parasites and not discrete clusters as we would expect if there were “abrupt” changes in expression. The heatmap shown in their Figure 4 also seems to indicate that each gene is not either on or off but gradually increasing or decreasing in expression (though we would agree that this is quite subjective). The three clusters of genes described in Reid et al., 2018 are different from what we observed in our data (e.g., our new Figures 2A, 3 and 4) and we wonder if this could be caused by an uneven distribution of the parasites along the intraerythrocytic cycle in the Reid study which could introduce some apparent clustering. We have included a comparison between our data and the recent P. berghei data generated by Howick et al. with the same technology as used in our study. This analysis highlights the similarities in transcriptional changes between these species along the intraerythrocytic cycle (see e.g., Figure 1B) and, we think, the conserved continuous changes in gene expression throughout the parasites’ development. We have also included a short discussion on this aspect on pages 13-14.**

Although the authors have included a comparison with the *P. berghei* data from Howick et al, as well as some technical comparisons with other single-cell plasmodium data sets, they do not address what is differentially expressed over development in a species-dependent manner, *i.e.* do the same genes show the same pattern of expression across both Pb Pf, and Pv? The comparison of the shape of the PCA is a very superficial level look at similarity in expression, it is helpful to have as a figure, but more needs to be done, especially given the new title stating the paper is about species-specific expression profiles.

Reviewer 3

Page 2 line 17- Was there any way for the authors to look at their parasites microscopically before generating the material for an RNA-sequencing library (as in reference 29)? How robust is the pseudotime data reduction i.e. can the age and life stage of parasites analyzed in this manuscript be said to be known with absolute certainty?

**We prepared blood smears from each sample and counted an estimated 10,000 red blood cells prior to processing through MACS columns. We agree that it would have been better to also have a second smear after MACS enrichment, but we did not have enough sample from the column. We have now included in Table 1 the stage composition determined by blood smear from each sample (prior to MACS enrichment).**

**Regarding the calculation of the pseudotime, we believe that this metric captures most of the variation occurring along the developmental cycle. The absolute value of this estimate is probably not very informative (as it depends on changes in gene expression and on the distribution of the parasites along the intraerythrocytic cycle) but the ranking reflects morphological changes in the parasites, appears robust with regards to the data cutoffs and normalization (see above), and is correlated with the gene expression variation observed in P. berghei.**

Concerns raised by reviewer 3 could be alleviated by correlating expression of the single-cell transcriptomes to those of ex vivo Pv bulk data. This would provide more insight into what the pseudotime values actually correspond to in real time. If the data does not correlate well with bulk, the authors have outlined a clear reason (lack of synchronicity or washed out by trophs) above that would be an interesting discussion point.

In keeping with the above content, can you use the existing Pv gene models to predict DNA cis-regulatory motifs based on the temporal profile of mRNA abundance? This would provide a very convincing argument that the precisely regulated timing of transcription factor expression is relevant for controlling the rest of the transcriptome, as the authors have suggested.

**We tried to identify clusters of co-expressed genes (using a modified version of WGCNA) to then search for enriched motifs in the 5’ region of each gene but failed to obtain convincing modules (probably due to the high number of drop-offs). At this point, we feel that our current data are not sufficient to address this question.**

There are many other methods to cluster genes besides WGCNA that might provide more clarity into patterns of coexpression. Saelens et al 2018 is a good review of the many different options (<https://www.ncbi.nlm.nih.gov/pubmed/29545622>). This could provide clusters that would allow for the identification of enriched motifs. Additionally, if greater than 40% of transcription is not assigned to a gene, then assigning regulatory regions will be a difficult task, so further clarification in the manuscript about genes in particular is required. If the annotations are so poor still that genes cannot be assigned, then this needs to be far clearer throughout the manuscript and explored. It does not mean that the results are not sound, but the “windows” based approach is confusing and needs to be more clearly explained why it is necessary and where possible, demonstrate that it works well (e.g., when windows can be clearly assigned to genes, the genes make sense).
