# Supplementary material for: Single-cell transcription analysis of Plasmodium vivax blood-stage parasites identifies stage- and species-specific profiles of expression
Source: PLoS Biol. 2020 May 4;18(5):e3000711. doi: 10.1371/journal.pbio.3000711 (PMC7224573; doi:10.1371/journal.pbio.3000711)
Supplement: Attachment — Submitted filename: Responses_030720.docx [file pbio.3000711.s052.docx]

**We would like to thank the academic editor and the reviewers for their quick review of our revised manuscript. We apologize for the delay in submitting this new revision: to address the remaining concerns of reviewer 2 and provide additional support for our interpretations, we found that extra time was needed to download, process, and analyze the raw sequence data from the Howick *et al.* study, steps that were not trivial. The results described below are now incorporated into the manuscript (taking into account the comments from the academic editor indicated in blue).**

Reviewer 2:

The improved method sections provides clarity on why this bespoke analysis was performed; however, it would still increase the impact of the paper if there was a more in depth comparison between the CellRanger output and the window based count table. How much of the biology that is discussed in the manuscript is actually lost by using CellRanger? Should all Plasmodium RNA-seq studies on P. vivax use this new method from now on? The authors should provide at least one quantitative comparison of results using their bespoke method and CellRanger with UTRs extended in the GTF by a reasonable amount. This can be supplementary, and their main text can stay as it is, but it is important to do this.

**Our Academic Editor suggests that you have mounted a convincing case as to why you need to use your bespoke method.**

The new Figure S2 appears to indicate that >40% of observed transcription is more than 2.5kb away from an annotated gene. Is this true? It would be important to further discuss this in the manuscript if so, and draw comparisons with results from bulk RNAseq. Additionally, for the remaining windows that are within 1kb of a gene, it is important to do a more clear-cut gene-for-gene comparison to the other species scRNAseq data out there, as noted in the next section's comment.

**Our Academic Editor indicates that this concern needs to be addressed and agrees with the reviewer that the bulk RNAseq should reveal roughly the length of 3’ UTRs which would validate your windows approach.**

**The reviewer brings an important point, although the number cited is not entirely correct: as indicated on Figure S2, 40% of the windows containing scRNA-seq reads are located more than 2.5 kb downstream or more than 2 kb upstream of the annotated 3’-end of a gene (which could still be within the gene). We show below the distribution of the number of 10X reads according to the closest gene, both for our *P. vivax* data and the *P. berghei* data generated by Howick *et al.* using the same technology. These data also address the previous point of the reviewer and suggest that, even for *P. berghei*, a significant amount of valuable and important 10X data is discarded if the analyses are restricted to the current gene annotations.**

|  | **Number of reads mapped to each 500-bp windows based on its proximity to the closest 3’-end.** “0” corresponds to a window containing an annotated 5’-end, “1”-“5” indicate windows located 1-5 windows of 500 bp downstream of the annotated 5’-end, and “-2”/”-1” upstream of the 5’-end. “X” represents bins located outside of these regions. See legend of Figure S2 for details. The *P. vivax* data is shown in orange bars, the *P. berghei* data from *Howick et al.*, in blue. |
| --- | --- |

**Using bulk RNA-seq to confirm that the scRNA-seq peaks indeed correspond to unannotated UTRs is not trivial. Previous *P. vivax* bulk RNA-seq data were derived from whole blood, and transcripts from trophozoites were likely overrepresented to the detriment of other stages (Kim *et al.*, 2019). In addition, the scRNA-seq peaks located in the body of annotated genes (but not close to the 3’-end as expected) cannot be globally validated (or rebutted) on the basis of those data. Nevertheless, we present representative examples of bulk RNA-seq data from patient samples supporting our scRNA-seq analyses and identifying the presence of unannotated or misannotated transcripts in the genome.**

**Examples of bulk RNA-seq data supporting signals of scRNA-seq in regions where annotations are incomplete.** For each region, the top-most track shows bulk RNA-seq data from one Cambodian patient and the next tracks, scRNA-seq data from 12 individual parasites. Current gene annotations are shown by the blue bars at the bottom. **(A)** and **(B)** screenshots showing regions where bulk RNA-seq and scRNA-seq concordantly indicate the presence of unannotated transcripts in gene “deserts”. **(C)** Example of an unannotated transcript located immediately after one annotated transcript.

**We have also generated preliminary data using long read sequencing (PacBio) following 10X single cell mRNA library preparation. We are currently working on how to best analyze these data systematically, but we include below representative examples demonstrating the incomplete annotations of *P. vivax* genes and the common occurrence of antisense unannotated transcripts (which contributes to the number of scRNA-seq peaks mapping outside “genes”).**

**Examples of incompletely annotated transcripts supported by long read sequencing.** Each panel shows the sequences obtained from all individual parasites at a given locus (in aggregates) after 10X library preparation and sequencing using PacBio or Illumina. The dark blue bars indicate the current *P. vivax* gene annotations in PlasmoDB and the lighter bars below, our predicted isoforms based on long read sequencing. Reads and annotations colored in blue are derived from the “plus” strand, those in red from “minus” strand. **(A)** The two peaks observed by Illumina sequencing, neither aligning with the 3’-end of the annotated gene, correspond to two different isoforms of a two-exon gene. **(B)** The two peaks observed in the Illumina data (one being a composite of reads from the “plus” and “minus” strands) actually derive from two isoforms of the annotated gene (with an extended 5’-UTR and variable length at the 3’ end) and one short antisense transcript overlapping with the 3’-end of the long isoform. A full length antisense transcript seems to account for a fourth, minor, transcript. **(C)** A more complicated gene with multiple isoforms (primarily differing in their 3’ end) and, again, some antisense transcripts. Note that these examples are not cherry-picked exceptions but reflect the diversity of isoforms observed at many (most?) genes (with the caveat that these data have only been preliminarily analyzed).

**To summarize, the scRNA-seq peaks and their “distant” locations with regards to annotated 3’-end of *Plasmodium* genes are genuine: we observed the same patterns in *P. vivax* and *P. berghei*; these peaks are shared across parasites; and they are consistent with bulk RNA-seq and long read sequencing data. We acknowledge that this response to the reviewer’s and editor’s comments involves specific examples instead of a more global treatment, but implementing a systematic reannotation and validation of gene isoforms in the *P. vivax* genome is going to be a major effort beyond the scope of the present report. Our analyses focus on global differences in expression between parasites and are not influenced by the “annotation” of these signals. Interpretation of some specific findings may vary if the signals are associated to the wrong genes, and we do caution the readers about gene-based interpretations on page 5. Overall, our study goes beyond state-of-the-art treatments that rely on currently-available annotations which, if indiscriminately applied, may fail to recognize (or even collapse) the signals from different transcripts.**

A better way to display information currently contained within S2 would be a conventional coverage plot across all genes including multiple windows beyond the stop codon. If the authors think the unannotated transcripts that are more than 500 bp from an annotated gene are driving the biology in their data, more work needs to be done on when and where they are expressed. It would be reassuring to see that the reads that map to these new 3' UTR regions in Pv map to 3' UTRs in orthologous genes.

**Academic Editor suggests that if orthologous means the 3’ UTRs in scRNAseq in other plasmodium spp, this wouldn't be very informative as the 3’ UTRs may be of different lengths. Another interpretation is the reviewer meant paralogs within Pv; finally they might mean the same genes in other Pv transcriptomes; the last interpretation seems reasonable but seems to reiterate the comment in the previous paragraph.**

Although the authors have included a comparison with the P. berghei data from Howick et al, as well as some technical comparisons with other single-cell plasmodium data sets, they do not address what is differentially expressed over development in a species-dependent manner, i.e. do the same genes show the same pattern of expression across both Pb Pf, and Pv? The comparison of the shape of the PCA is a very superficial level look at similarity in expression, it is helpful to have as a figure, but more needs to be done, especially given the new title stating the paper is about species-specific expression profiles.

**Academic Editor agrees that the PCA plot is suggestive of similarity of profile but is not informative of the number or identity of similarly regulated genes across the life cycle. The new discussion and your claims of a gradual progression through transcription are useful, but our Academic Editor indicates that some more detailed comparison with other species is warranted.**

**The PCA analysis is based on the gene expression profile of each individual parasite and the mirrored trajectories of the *P. berghei* and *P. vivax* blood stage parasites do therefore indicate that the same genes show the same change in expression during the IEC: the same eigenvectors explain the differences between, for example, trophozoites and schizonts of both *P. vivax* and *P. berghei* and therefore this development is regulated by the same genes (those contributing to these eigenvectors). In-depth analysis of the similarities in expression between species is also presented in the study by Howick *et al*. Our statement on the “species-specific profiles of expression” is meant to highlight our findings on the variation of the proportion of mRNAs derived from genes without orthologs in other *Plasmodium* species. We think this is an interesting and novel finding worth emphasizing but would remove it from the title if the reviewer and academic editor consider it misleading.**

**Reviewer 3:**

Concerns raised by reviewer 3 could be alleviated by correlating expression of the single-cell transcriptomes to those of ex vivo Pv bulk data. This would provide more insight into what the pseudotime values actually correspond to in real time. If the data does not correlate well with bulk, the authors have outlined a clear reason (lack of synchronicity or washed out by trophs) above that would be an interesting discussion point.

**Academic Editor isn't convinced that this comparison would be useful to address this issue. Pseudotime has been accepted widely as a measure and, as the reviewers acknowledge, a disparity between pseudotime here and bulk RNAseq elsewhere would not reveal whether the pseudotime or the bulk RNAseq were incorrect and thus would not be very useful.**

There are many other methods to cluster genes besides WGCNA that might provide more clarity into patterns of coexpression. Saelens et al 2018 is a good review of the many different options (<https://www.ncbi.nlm.nih.gov/pubmed/29545622>). This could provide clusters that would allow for the identification of enriched motifs. Additionally, if greater than 40% of transcription is not assigned to a gene, then assigning regulatory regions will be a difficult task, so further clarification in the manuscript about genes in particular is required. If the annotations are so poor still that genes cannot be assigned, then this needs to be far clearer throughout the manuscript and explored. It does not mean that the results are not sound, but the "windows" based approach is confusing and needs to be more clearly explained why it is necessary and where possible, demonstrate that it works well (e.g., when windows can be clearly assigned to genes, the genes make sense).

**Academic Editor believes that this reiterates the reviewer's concerns re the validity of the windows approach and suggests that you address this by comparing your RNAseq to Pv bulk RNAseq to confirm the validity of the predicted 3’ UTR length.**

**Academic Editor:**

No fig legends for figs s7 and s8

**These are now included in the Supplemental Material files.**

The authors meaning behind the lack of co-variation between male and female gam gene expression is now clearer that they are not using words with mathematical meaning in a non-mathematical fashion but I’m not convinced that the data supports the conclusion. I agree that, for example, high levels of female gam gene expression can occur with low, high or medium levels of male gam gene expression but I don’t see why this would necessitate separate processes of gametocytogenesis. An alternative explanation consistent with the data is a common pathway of sexual differentiation that defaults to one sex, then those that fail to switch to the other sex will end up the default sex, the proportion that fail to switch can vary but the processes are not separate. I don’t suggest that this actually happens but nor do I think the presented evidence confirms separate processes of gametocytogenesis. The author’s intended meaning is still somewhat opaque in the use of the word “terminal”.

Gametocytogenesis is a terminal process, it is unclear whether terminal encompasses the entire process (as I assumed above) or merely the later stages when gametocyte sex can be established. If the latter, then the observation is facile that further male and female differentiation are separate processes after male and female sex has been established. If gametocytogenesis is proposed to be sexually divergent from initiation then there is some implicit difference that precedes or is concurrent with AP2G initiating commitment, is there any evidence for such a difference? I don’t think the authors provide any evidence of such a mechanism and I think this section should be redrafted much more cautiously and the actually intended meaning conveyed much more explicitly. If this section is just speculation then comments re “confirming” separate processes of gametocytogenesis should be removed.

**Thanks for this detailed and informative comment. We agree with the suggestion and have removed the statement on the gametocytogenesis on page 12.**

My comments re the observed increase in H4 expression following Cq treatment and the comments on H3.3 were not meant to imply that the paper lacked a discussion of histone gene expression but rather to indicate that increased nucleosome density as a resistance mechanism was not the only possible explanation for altered H4 expression levels, increased H3.3-H4 deposition would alter the composition but not the density of nucleosomes. I don’t suggest that the authors need to address H3.3 but they have now modified the MS to include a fairly long section on general histone gene expression and the expression of several chromatin regulatory proteins during the Pv lifecycle as revealed by the scRNAseq. Is this novel and noteworthy? The transcriptional profile of Pf histone genes has been long known from bulk transcriptomes.

**We also included this section as a response to the previous round of comments that brought up the lack of morphological characterization of the parasites and reference “anchors” for interpreting the gene expression profiles. Since the expression profiles of histones are fairly well-characterized and easy to interpret with regards to the changes in DNA in dividing parasites, we thought these data were a simple way to demonstrate the validity of our findings. The variation in histone expression also illustrates, in a more convincing manner than the heatmap, the gradual changes in expression during the parasite development. We would therefore prefer to keep this paragraph in the manuscript.**

The authors table s3 shows that nearly all of the sex-specific genes identified in Pv scRNAseq in this study were also found in Pb by Yeoh et al using bulk rnaseq of sex specific separated gams also many were shown by obaldia and marti. All of these papers should be cited in the main body of the MS and included in the text not just the supplement. The authors point out the majority of the female gam genes they describe are restricted to this stage and sex but many of these genes are present in male and female Pb gams according to table s3, this should be commented on, is this a genuine species difference or possibly a technical artefact?

**The references and a short comment on the discrepancy with the Yeoh study are now included on page 12.**
