# Supplementary material for: Single-cell transcription analysis of Plasmodium vivax blood-stage parasites identifies stage- and species-specific profiles of expression
Source: PLoS Biol. 2020 May 4;18(5):e3000711. doi: 10.1371/journal.pbio.3000711 (PMC7224573; doi:10.1371/journal.pbio.3000711)
Supplement: Attachment — Submitted filename: Response.docx [file pbio.3000711.s053.docx]

**We have addressed the remaining comments from the academic editor and included the results of the last requested analysis in the manuscript (see also below). Thank you again for the thorough review and the constructive comments and suggestions that have significantly improved the manuscript.**

Comments from the Academic Editor:

The authors have largely addressed the queries raised by the reviewer and myself (editor).

Fig s2 is difficult to find in the attached revised MS, it is in suppl materials but its presence there is not indicated in the main MS, the suppl materials file could be renamed to indicate that it contains many of the suppl figs. I assume the legend in the response and fig s2 uses 5’-end in error when the authors mean 3’-end? If so, then I think this data is convincing that approx. half the reads are mapping within 500 bp of the end of the gene.

**Figure S2 is now cited in the Material and Methods section as well as in the modified Results section (page 9). The legend of Figure S2 was indeed incorrect and should have referred to the 3’-end as noted by the editor. Thank you for noticing this mistake. We have fixed the legend accordingly.**

The authors also show convincing examples of incorrect gene annotations which could explain the distant locations of their scRNAseq reads from the annotated gene 3’ ends.

However, the authors don’t perform the requested broad comparison with bulk rnaseq because; “implementing a systematic reannotation and validation of gene isoforms in the P. vivax genome is going to be a major effort beyond the scope of the present report.” Maybe the authors have misunderstood the requested, trivial comparison to bulk RNAseq. The intention of the requested analysis was to distil multiple comments of R2 to a single validation of the assignment of reads to transcripts and genes. This could be addressed by comparing a table of bulk RNAseq assembled transcripts, eg from cufflinks, to the scRNAseq. This would not require any comparison with existing annotations. A simple bedtools intersect of the concatenated scRNAseq consensus peak coordinates and the bulk RNAseq transcripts should reveal the proportion of scRNAseq “peaks” that are corroborated by bulk RNAseq transcripts. The authors already have the bam files for the bulk rnaseq so this comparison would be trivial and would allay concerns that the scRNAseq was incorrectly assigning reads to distant genes.

**We have now conducted the analysis suggested by the reviewer, though using stringTie, the newer equivalent of cufflinks. Briefly, we generated *de novo* transcripts using bulk RNA-seq data from one *P. vivax*-infected Cambodian patient and analyzed the positions of the scRNA-seq reads with regard to the 3’-end of these transcripts. 60,785,866 reads (34%) mapped to the last 500 bp of the 3’-end of annotated genes, based on PlasmoDB, which represents a 2.5-fold enrichment over what would be expected solely by chance (given the number of annotated transcripts and the size of the *P. vivax* genome). When we conduct the same analysis using the *de novo* transcripts determined from bulk RNA-seq, 38,943,973 reads (22%) map to the last 500 bp of the 3’-end of these predicted transcripts, representing a 3.4-fold enrichment over what is expected by chance, demonstrating that many of the scRNA-seq signals do indeed overlap with unannotated 3’-gene ends. Overall, 45% of all scRNA-seq reads map to the last 500 bp of the 3’-end of either an annotated or predicted transcript. A short description of these analyses are presented on pages 9 and 10, and the methods are described on pages 5-6.**

I understood the reviewer to have agreed that the PCA plot shows that the variation in transcriptome of the approx. 5000 genes through development is largely concordant hence the similar eigenvectors resulting in a similar PCA. However, there will be some genes that are differently expressed between species across development. These are the genes that I understood the reviewer wanted discussed. However, upon re-reading the MS I think this additional discussion is probably unnecessary.

The authors have satisfactorily addressed all of the other comments and queries from the editor and reviewer.

**Thank you again for your help and suggestions.**
